# SOCS3 Negatively Regulates Cardiac Hypertrophy via Targeting GRP78-Mediated ER Stress During Pressure Overload

**DOI:** 10.3389/fcell.2021.629932

**Published:** 2021-01-26

**Authors:** Shuang Liu, Wen-Chang Sun, Yun-Long Zhang, Qiu-Yue Lin, Jia-Wei Liao, Gui-Rong Song, Xiao-Lei Ma, Hui-Hua Li, Bo Zhang

**Affiliations:** ^1^College of Basic Medical Sciences, Dalian Medical University, Dalian, China; ^2^Department of Microbiology, College of Basic Medical Sciences, Dalian Medical University, Dalian, China; ^3^Beijing Key Laboratory of Cardiopulmonary Cerebral Resuscitation, Department of Emergency Medicine, Beijing Chaoyang Hospital, Capital Medical University, Beijing, China; ^4^Department of Cardiology, Institute of Cardiovascular Diseases, First Affiliated Hospital of Dalian Medical University, Dalian, China; ^5^Department of Health Statistics, School of Public Health, Dalian Medical University, Dalian, China

**Keywords:** cardiac hypertrophy, heart failure, socs3, glucose regulatory protein 78, endoplasmic reticulum stress

## Abstract

Pressure overload-induced hypertrophic remodeling is a critical pathological process leading to heart failure (HF). Suppressor of cytokine signaling-3 (SOCS3) has been demonstrated to protect against cardiac hypertrophy and dysfunction, but its mechanisms are largely unknown. Using primary cardiomyocytes and cardiac-specific SOCS3 knockout (SOCS3cko) or overexpression mice, we demonstrated that modulation of SOCS3 level influenced cardiomyocyte hypertrophy, apoptosis and cardiac dysfunction induced by hypertrophic stimuli. We found that glucose regulatory protein 78 (GRP78) was a direct target of SOCS3, and that overexpression of SOCS3 inhibited cardiomyocyte hypertrophy and apoptosis through promoting proteasomal degradation of GRP78, thereby inhibiting activation of endoplasmic reticulum (ER) stress and mitophagy in the heart. Thus, our results uncover SOCS3-GRP78-mediated ER stress as a novel mechanism in the transition from cardiac hypertrophy to HF induced by sustained pressure overload, and suggest that modulating this pathway may provide a new therapeutic approach for hypertrophic heart diseases.

## Introduction

Pathological cardiac hypertrophy is typically characterized by increased cardiac myocyte cell size, interstitial fibrosis, myocyte apoptosis, and contractile dysfunction (Heineke and Molkentin, 2006; Nakamura and Sadoshima, 2018). Various forms of stress or injury, such as hypertension, valve disease, and ischemic heart disease, induce cardiac hypertrophy and heart failure (HF) through multiple mechanisms, including abnormal signal transduction, disrupted intracellular calcium handling, imbalance between protein synthesis and degradation, endoplasmic reticulum (ER) stress, and mitochondrial dysfunction. The ER is a critical organelle involved in intracellular protein synthesis, folding, and translocation, as well as calcium homoeostasis (Gotoh et al., 2011; Rashid et al., 2015). Recent evidence revealed that ER stress is associated with various heart diseases, such as ischemic heart diseases, cardiac hypertrophy, and HF (Yamaguchi et al., 2003; Fu et al., 2010; Minamino and Kitakaze, 2010; Yao et al., 2017). Thus, improved understanding of the regulatory mechanisms of ER stress in heart disease will facilitate identification of potential targets for intervention.

The suppressor of cytokine signaling (SOCS) family of proteins includes eight intracellular proteins, SOCS1–7 and cytokine-inducible SH2 protein (CIS) (Yasukawa et al., 2012), which are structurally characterized by a variable N-terminal region, a central SH2 domain, and a C-terminal SOCS box motif (Masuhara et al., 1997; Hilton et al., 1998). Among them, CIS and SOCS1–3 proteins directly interact with gp130 and/or Janus kinases (JAKs), thereby inactivating downstream mediators. Gp130, a common β-receptor component of the IL-6 family of cytokines, regulates three downstream signaling pathways in cardiomyocytes, JAK/STAT, mitogen-activated protein kinase, and phosphoinositide 3 kinase (PI3K/AKT), which play a pathological role in the development of cardiac hypertrophy and HF after various stimuli, such as leukemia inhibitory factor (LIF) and pressure overload (Hirota et al., 1995; Kunisada et al., 1998; Uozumi et al., 2001; Yasukawa et al., 2001, 2012; Fischer and Hilfiker-Kleiner, 2007). Notably, SOCS3-knockout mice are embryonic-lethal as a result of placental deficiency and marked erythrocytosis (Marine et al., 1999; Roberts et al., 2001). Moreover, SOCS3 is a mechanical stress-inducible gene that is markedly upregulated in hypertrophic hearts after 2 weeks of transverse aortic constriction (TAC) (Yasukawa et al., 2001). However, clinical studies have shown reduced SOCS3 expression in failing human myocardium, where it plays a critical role as a negative feedback regulator of JAK-mediated gp130 signaling (Podewski et al., 2003; Margulies et al., 2005; Mann et al., 2010). Indeed, cardiac-specific knockout of SOCS3 (SOCS3cko) results in cardiac hypertrophy, chamber dilatation, and dysfunction accompanied by activation of gp130 signaling and abnormal myofilament Ca2^+^ sensitivity after pressure overload (Yajima et al., 2011). Conversely, adenovirus-mediated overexpression of SOCS3 in cardiomyocytes markedly inhibits the LIF and CT-1-induced hypertrophic response, as well as activation of gp130 downstream signals (Yasukawa et al., 2001), suggesting that SOCS3 may be a new potential therapeutic target for treatment of cardiac hypertrophy and HF. Although the role of SOCS3 regulation on cardiac gp130 signaling in pressure overload has been relatively well-evaluated, little is known about whether other regulatory mechanisms are involved in the action of SOCS3 in cardiac function.

In this study, using primary cardiomyocytes, cardiac-specific knockout of SOCS3 (SOCS3cko) or overexpression mice infected with rAAV9-SOCS3, we found that SOCS3-GRP78-ER stress signaling was essential for the transition from cardiac hypertrophy to HF during pressure overload, we found that SOCS3 acts as a negative regulator of cardiac hypertrophy and dysfunction induced by pressure overload by targeting GRP78 for ubiquitination and degradation by the proteasome. Thus, we demonstrated that SOCS3-GRP78-ER stress signaling was essential for the transition from cardiac hypertrophy to HF during pressure overload, and suggest that SOCS3 may represent a potential therapeutic target for treating hypertrophic heart diseases.

## Materials and Methods

### Animal Models and Treatment

Wild-type (WT) and SOCS3-flox (SOCS3f/f) mice were obtained from Jackson Laboratories (Bar Harbor, ME). Cardiac-specific knockout of SOCS3 (SOCS3cko) mice were generated by mating SOCS3f/f mice with mice expressing Cre recombinase under the α-myosin heavy chain (α-MHC) promoter as described previously (Oba et al., 2012). All animals were C57BL/6J background. To induce Cre-dependent recombination, tamoxifen (20 mg/kg body weight, Sigma-Aldrich) was injected intraperitoneally for 5 days over a 3-week duration before experiments. SOCS3f/f mice were used as a control for SOCS3cko mice. Male mice (aged 8–10 weeks) were maintained in a pathogen-free facility at the Laboratory Animal Center at Dalian Medical University. All procedures were performed in accordance with protocols outlined in the Guide for the Care and Use of Laboratory Animals published by the US National Institutes of Health (NIH publication No. 85-23, revised 1996) and approved by the Committee on the Ethics of Animal Experiments of Dalian Medical University, as described in our previous study (Xie et al., 2018).

Male SOCS3f/f and SOCS3cko mice were anesthetized with isoflurane and subjected to pressure overload induced by transverse aortic constriction (TAC) for 4 weeks, as previously described (Li et al., 2007; Xie et al., 2019). Sham-treated mice underwent the same operation without aortic constriction. After recovering from surgery, mice were injected intraperitoneally with 4-PBA (20 mg/kg/day) or vehicle (dimethyl sulfoxide, DMSO) daily for 4 weeks. 4-PBA was first dissolved in DMSO, then diluted in 0.9% NaCl (Metz et al., 1977).

### Histopathological Analysis

Heart samples were quickly dissected out and rinsed with cool sterile saline, and then fixed in 10% paraformaldehyde, embedded in paraffin, and cut into 5-μm-thick sections for histological analysis. Heart sections were stained with hematoxylin and eosin (H&E), wheat germ agglutinin (WGA) and Masson's trichrome as previously described (Wang L. et al., 2018; Xie et al., 2018). All digital images were taken at ×100 or ×200 magnification of 15–20 random fields from each heart sample. Analysis of myocyte cross-sectional area was calculated by measuring 150 to 200 cells per slide. The areas of myocardial fibrosis were evaluated by Image Pro Plus 3.0 (Nikon, Japan).

### Proteomic Analysis

Primary experimental procedures for proteomic analysis included protein extraction, trypsin digestion, high-performance liquid chromatography fractionation, liquid chromatography with tandem mass spectrometry, and data analysis supported by Jingjie PTM BioLabs (Hangzhou, China).

### Statistical Analysis

All data are expressed as mean ± SEM. All statistical analyses were performed with SPSS 16.0 software (IBM, Armonk, NY). For two-group comparisons, we performed Student's *t*-test. For comparison of multiple groups, significance was determined using 1-way or 2-way ANOVA with Tukey's *post-hoc* test. *P* < 0.05 was considered statistically significant.

## Results

### SOCS3 Overexpression Inhibited Cardiomyocyte Hypertrophy and Activation of Gp130 Signaling *in vitro*

**Figure 1:**
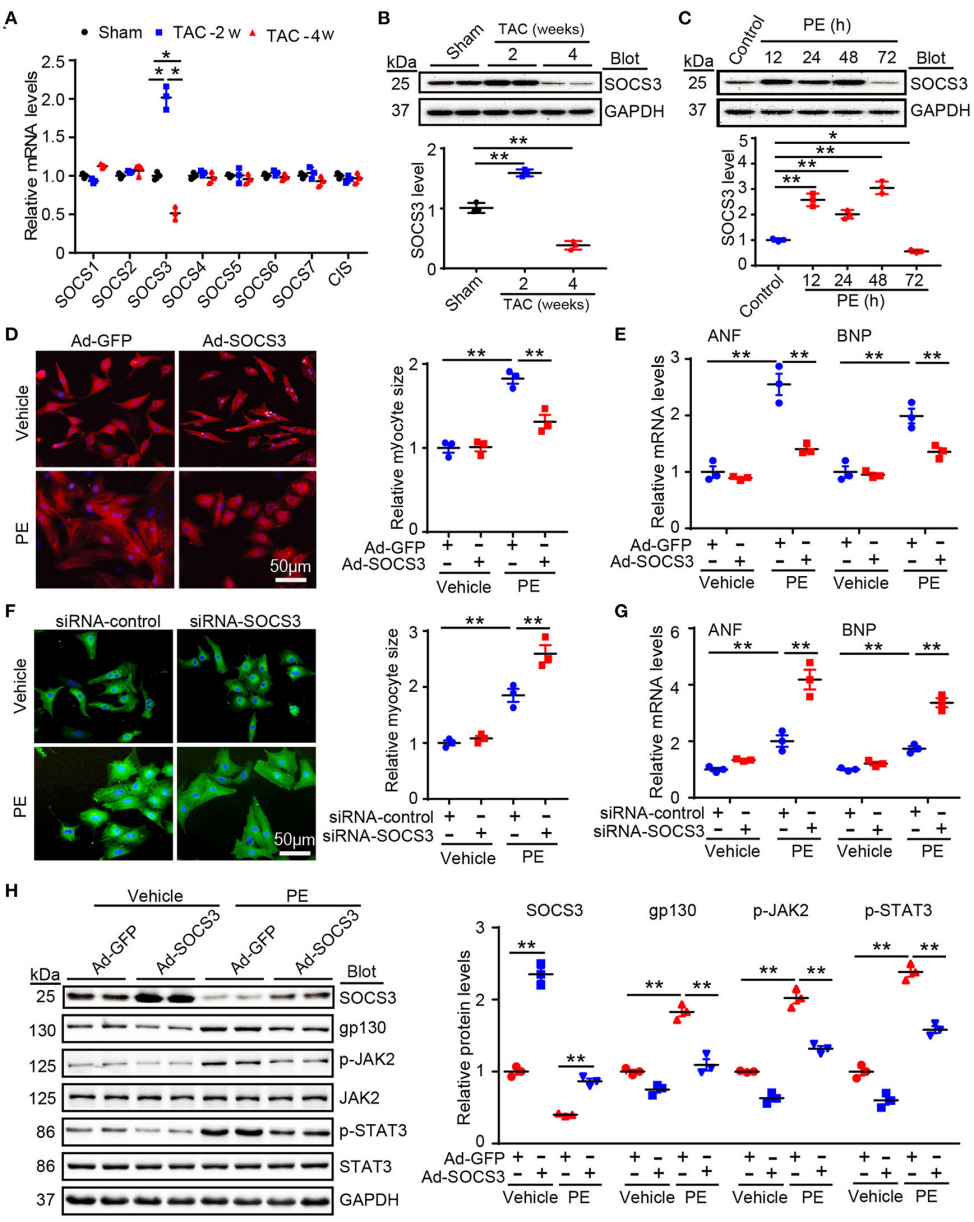
SOCS3 regulated cardiomyocyte hypertrophy and gp130 signaling *in vitro*. **(A)** qPCR analyses of mRNA levels of eight SOCS family members, including SOCS1–7 and CIS, in hearts of Sham and TAC-treated mice at 2 and 4 weeks (*n* = 3 per group). **(B)** Immunoblotting analysis of SOCS3 protein levels in hearts of Sham and TAC-treated mice at 2 and 4 weeks (upper), and quantification (lower, *n* = 3 per group). **(C)** Immunoblotting analysis of SOCS3 protein levels in neonatal rat cardiomyocytes (NRCMs) exposed to PE (100 μmol/L) for different durations (upper), and quantification (lower, *n* = 3). **(D)** Images of double immunostaining (red indicates α-actinin, blue indicates DAPI-stained nuclei) of NRCMs infected with Ad-GFP control or SOCS3 after 72 h of PE treatment (left). Quantification of cell surface area (right, 150 cells counted per experiment, *n* = 3). Scale bar = 50 μm. **(E)** qPCR analyses of atrial natriuretic factor (ANF) and brain natriuretic peptide (BNP) mRNA expression in each group (right, *n* = 3). **(F)** Images of double immunostaining (green indicates α-actinin, blue indicates DAPI) of NRCMs infected with siRNA-control or siRNA-SOCS3 after 72 h of PE treatment (left). Quantification of cell surface area (right, 150 cells counted per experiment, *n* = 3). Scale bar = 50 μm. **(G)** qPCR analyses of ANF and BNP mRNA expression (right, *n* = 3). **(H)** Immunoblotting analysis of SOCS3, gp130, p-JAK2, JAK, p-STAT3, STAT3, and GAPDH protein levels in NRCMs infected with Ad-GFP control or SOCS3 after 72 h of PE treatment (left), and quantification (right, *n* = 3). Data are presented as mean ± SEM, and *n* represents number of samples per group. *P < 0.05, **P < 0.01.

To identify which SOCS family members are essential for cardiac hypertrophy and dysfunction, we first evaluated expression of endogenous SOCS members after stimulation with hypertrophic agonists. Quantitative real-time PCR analysis showed that among the eight SOCS family members, only SOCS3 was significantly upregulated at week 2 (the hypertrophic stage) and decreased at week 4 (the HF stage) after TAC (Figure 1A). This change in SOCS3 protein level was further validated by immunoblotting (IB) of the same hearts at different time points (Figure 1B). SOCS3 expression was also appreciably increased in neonatal rat cardiomyocytes (NRCMs) in response to phenylephrine (PE, 100 μmol/L) stimulation for 12–48 h, but was decreased at 72 h (Figure 1C). However, SOCS3 expression was not altered in neonatal rat cardiac fibroblasts after PE stimulation at different time points (Supplementary Figure 1A).

We next investigated the role of SOCS3 in cardiac hypertrophy *in vitro*. NRCMs were infected with an adenovirus overexpressing SOCS3 (Ad-SOCS3) or empty vector with green fluorescent protein (GFP, Ad-GFP), and treated with PE (100 μM) for 72 h. SOCS3 overexpression (increased by ~2-fold, Supplementary Figure 1B) significantly inhibited PE-induced increases of cardiomyocyte size and expression of the hypertrophic markers atrial natriuretic factor (ANF) and brain natriuretic peptide (BNP) (Figures 1D,E). Conversely, SOCS3 knockdown by siRNAs (decreased by ~50%, Supplementary Figure 1C) enhanced PE-induced hypertrophic responses compared with siRNA-control (Figures 1F,G). Accordingly, SOCS3 overexpression remarkably inhibited activation of the downstream targets gp130, p-JAK2, and p-STAT3 compared with the Ad-GFP control in NRCMs after PE treatment (Figure 1H). Overall, these results indicated that SOCS3 exerts an antihypertrophic role *in vitro*.

### SOCS3 Overexpression in Cardiomyocytes Prevented Cardiac Hypertrophy and Dysfunction Induced by Pressure Overload

**Figure 2:**
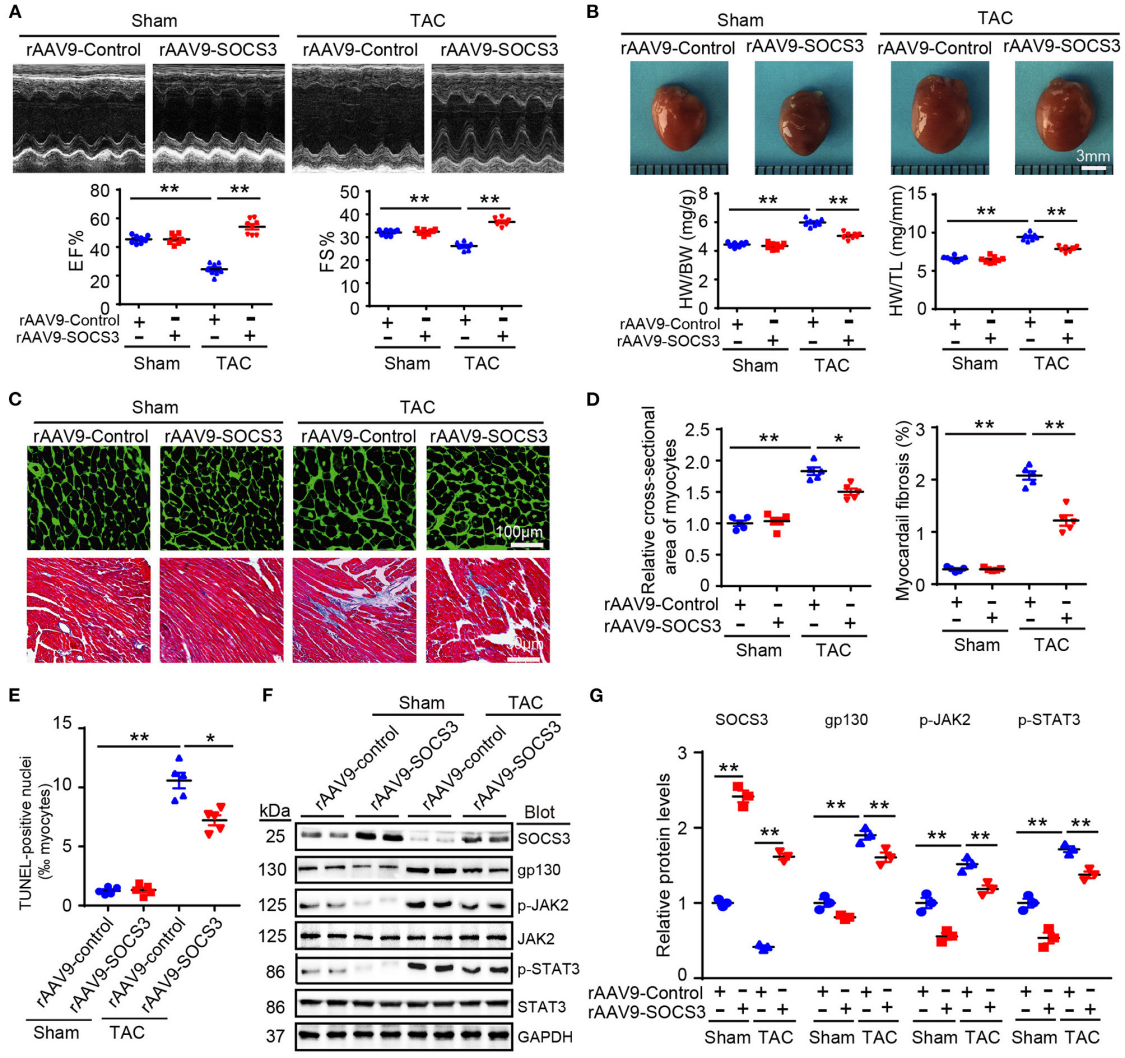
SOCS3 overexpression in cardiomyocytes attenuated TAC-induced cardiac hypertrophy in mice. Wild-type (WT) mice were injected with rAAV9-SOCS3 or rAAV9-Control for 3 weeks, and then subjected to Sham or TAC operation for 4 weeks. **(A)** M-mode echocardiography of the left ventricle (top). Measurement of ejection fraction (EF%) and fractional shortening (FS%) (bottom, *n* = 8). **(B)** Images of hearts for size measurement, as photographed with a stereomicroscope (top), as well as heart weight/body weight (HW/BW) and HW/tibial length (TL) ratios (bottom, *n* = 8). **(C)** Cardiac sections were stained by FITC-labeled wheat germ agglutinin (WGA), and myocardial fibrosis was detected by Masson's trichrome staining. Scale bar = 100 μm. **(D)** Quantification of relative myocyte cross-sectional area (200 cells counted per heart) and fibrotic area (*n* = 5). **(E)** Quantification of percentages of TUNEL-positive nuclei (*n* = 5). **(F)** Immunoblotting analysis of SOCS3, gp130, p-JAK2, JAK, p-STAT3, STAT3, and GAPDH levels in hearts. **(G)** Quantification of relative protein levels (*n* = 3). Data are presented as mean ± SEM, and *n* represents number of samples per group. *P < 0.05, **P < 0.01.

To examine the *in vivo* pathophysiological role of SOCS3 in the heart, we increased SOCS3 expression in WT hearts by injecting a rAAV9 expressing SOCS3 (rAAV9-SOCS3) or ZsGreen (rAAV9-ZsGreen, a negative control). Transfection efficiency and expression of SOCS3 were confirmed in hearts by fluorescence microscopy of ZsGreen (Supplementary Figure 2A), and immunoblotting analysis indicated a 2.3-fold increase of SOCS3 expression compared with control (Supplementary Figure 2B). Four weeks after TAC operation, echocardiographic assessment revealed that TAC significantly impaired contractile function, as reflected by decreased ejection fraction (EF%) and fractional shortening (FS%) in rAAV9-ZsGreen-injected mice compared with Sham groups, whereas rAAV9-SOCS3-injected mice recovered cardiac dysfunction similar to or better than Sham mice (Figure 2A). Furthermore, TAC-induced decompensation of hypertrophy, as indicated by increases in heart size, ratios of heart weight/body weight (HW/BW) and heart weight/tibia length (HW/TL), cross-sectional area of myocytes, and fibrotic area in rAAV9-ZsGreen-injected mice, were also significantly abrogated in rAAV9-SOCS3-injected mice (Figures 2B–D). Accordingly, mRNA expressions of ANF, BNP, and β-myosin heavy chain (β-MHC), collagen I, and collagen III were markedly reduced in rAAV9-SOCS3-infected mice compared with rAAV9-ZsGreen-infected animals after TAC (Supplementary Figures 2C,D). Moreover, SOCS3 overexpression markedly reduced TAC-induced cardiomyocyte apoptosis, as indicated by the number of TUNEL-positive nuclei in WT hearts compared with rAAV9-ZsGreen-infected hearts (Figure 2E). In addition, gp130, p-JAK2, and p-STAT3 protein levels were consistently downregulated in rAAV9-SOCS3-injected mice compared with rAAV9-ZsGreen-injected mice (Figures 2F,G). These results suggested that cardiac overexpression of SOCS3 enabled improvement of TAC-induced cardiac hypertrophy and dysfunction.

### Cardiac-Specific Ablation of SOCS3 Accelerated Pressure Overload-Induced Cardiac Hypertrophy and Dysfunction

**Figure 3:**
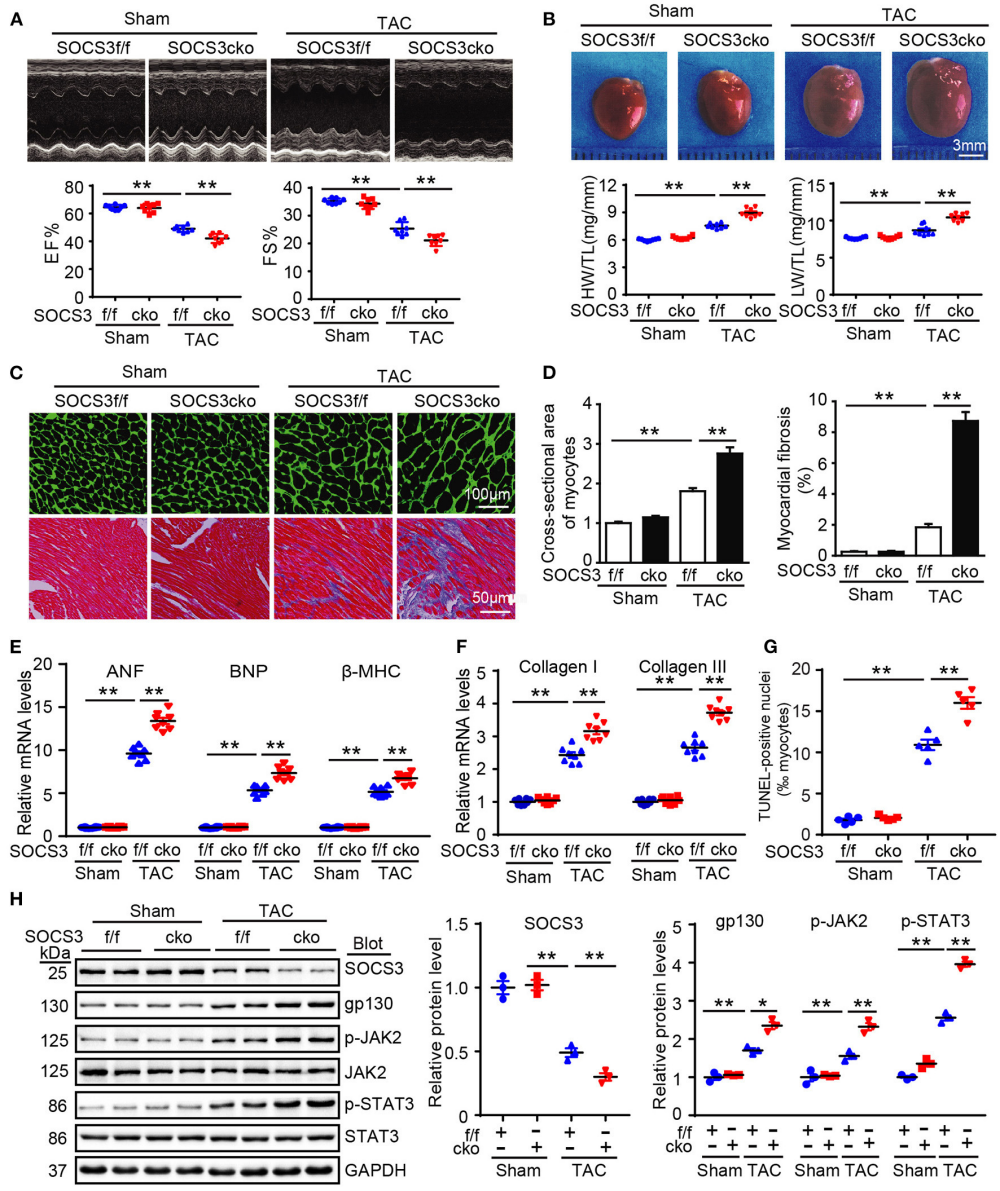
Ablation of SOCS3 in cardiomyocytes aggravated cardiac hypertrophy in mice after pressure overload. WT (SOCS3f/f) and cardiomyocyte-specific SOCS3 knockout mice (SOCS3cko) were subjected to Sham or TAC operation for 4 weeks. **(A)** M-mode echocardiography of the left ventricle (top). Assessment of EF% and FS% (bottom, *n* = 8). **(B)** Images of hearts for size measurement (top). HW/BW and HW/TL ratios (bottom, *n* = 8). **(C)** Cardiac myocyte size and fibrosis were examined by FITC-labeled WGA staining and Masson's trichrome staining, respectively. Scale bar = 100 μm. **(D)** Quantification of relative myocyte cross-sectional area (200 cells counted per heart) and fibrotic area (*n* = 5). **(E)** qPCR analyses of ANF, BNP, and β-myosin heavy chain (β-MHC) mRNA levels (*n* = 8). **(F)** qPCR analyses of collagen I and collagen III mRNA levels (*n* = 8). **(G)** Quantification of percentages of TUNEL-positive nuclei (*n* = 5). **(H)** Immunoblotting analysis of SOCS3, gp130, p-JAK2, JAK, p-STAT3, STAT3, and GAPDH protein levels in hearts, and quantification (*n* = 3). GAPDH was used as an internal control. Data are presented as mean ± SEM, and *n* represents the number of animals per group. *P < 0.05, **P < 0.01.

To precisely ascertain whether loss of SOCS3 in cardiomyocytes predisposes mice to HF, SOCS3f/f mice were bred with α-MHC-Cre mice to generate cardiomyocyte-specific SOCS3-knockout mice (SOCS3cko). Specific deletion of SOCS3 in cardiomyocytes was confirmed by immunoblotting analysis, as previously described (Yajima et al., 2011). Wild-type (SOCS3f/f) and SOCS3cko mice were subjected to Sham or TAC operation for 4 weeks. SOCS3f/f mice exhibited characteristics of HF, as indicated by significant reductions in EF% and FS% compared with Sham groups, and this effect was aggravated in SOCS3cko mice (Figure 3A). Moreover, SOCS3cko mice developed severe cardiac hypertrophy and fibrosis, as indicated by increased heart size, HW/TL and LW/TL ratios, cross-sectional area of myocytes, and interstitial collagen deposition compared with SOCS3f/f mice following TAC (Figures 3B–D). Similarly, the mRNA levels of ANF, BNP, β-MHC, collagen I, and collagen III were markedly upregulated in SOCS3cko mice compared with SOCS3f/f animals after TAC (Figures 3E,F). Loss of SOCS3 also induced cardiomyocyte apoptosis (increased number of TUNEL-positive nuclei) in SOCS3cko hearts compared with SOCS3f/f hearts after TAC (Figure 3G). Finally, the protein levels of gp130, p-JAK2, and p-STAT3 were significantly upregulated in SOCS3cko mice compared with SOCS3f/f mice after TAC (Figure 3H). There was no difference in these pathological parameters between the two groups after Sham operation (Figures 3A–H). These results demonstrated that SOCS3cko mice were more susceptible to TAC-induced hypertrophic remodeling and dysfunction.

### Loss of SOCS3 Caused Activation of ER Stress and Autophagy Leading to Mitochondrial Dysfunction

**Figure 4:**
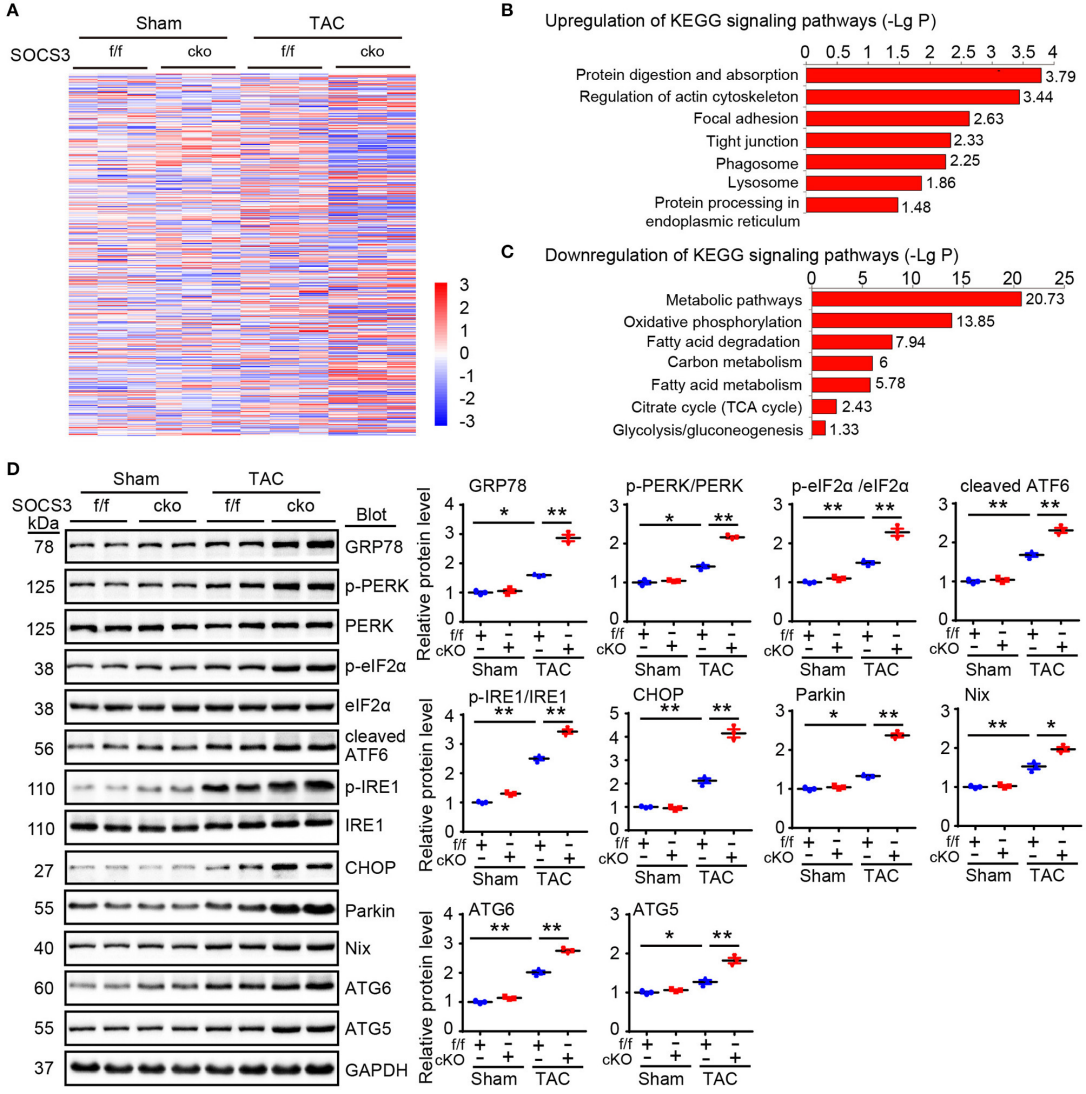
Proteomic analysis of heart tissues. **(A)** WT (SOCS3f/f) and cardiomyocyte-specific SOCS3 knockout mice (SOCS3cko) were subjected to Sham or TAC operation for 4 weeks. Proteomic analysis was performed using an iTRAQ-based strategy. The heat map of the protein expression differences from the proteomics between SOCS3f/f and SOCS3cko mice. The red color indicates upregulation and blue is for downregulation. **(B,C)** Analysis of Kyoto Encyclopedia of Genes and Genomes pathway enrichment for differentially expressed proteins in heart tissues. LgP represents the logarithm of *P*-value. **(D)** Immunoblotting analysis of GRP78, p-PERK, PERK, p-eIF2α, eIF2α, cleaved ATF6, p-IRE1, IRE1, CHOP, Parkin, Nix, ATG6, and ATG5 protein levels in hearts, and quantification (*n* = 3). GAPDH was used as an internal control. Data are presented as mean ± SEM, and *n* represents the number of animals per group. *P < 0.05, **P < 0.01.

Although ablation of SOCS3 in cardiomyocytes resulted in activation of gp130/JAK/STAT3 signaling leading to hypertrophic remodeling after long-term TAC (Yasukawa et al., 2001; Yajima et al., 2011), the underlying regulatory mechanism remains largely unknown. To identify novel targets or pathways involved in hypertrophic remodeling in SOCS3cko mice subjected to TAC, we performed proteomic analysis using an iTRAQ-based strategy (data are available via ProteomeXchange with identifier PXD014946). A total of 4,482 proteins were identified from these pairs of heart tissues, among which 3,622 proteins had quantitative changes (Figure 4A). Differentially expressed proteins were selected by filtering with an average cut-off of 1.5-fold change in expression and *p-*value ≤ 0.05 when comparing TAC-treated heart samples with their corresponding Sham tissues (Figure 4A). A total of 534 proteins qualified as differentially expressed between SOCS3f/f and SOCS3cko mice, including 297 upregulated and 237 downregulated proteins. Analysis of Kyoto Encyclopedia of Genes and Genomes (KEGG) pathway enrichment revealed that upregulated proteins in SOCS3cko mice were enriched for protein digestion and absorption, phagosomes and lysosomes, and protein processing in the endoplasmic reticulum (ER) (Figure 4B). Surprisingly, differentially expressed proteins that were downregulated were mostly localized to metabolic pathways and mitochondria. Indeed, 68 of 69 mitochondrial-related proteins were downregulated in SOCS3cko mice after TAC, including proteins linked to mitochondria-related functions such as oxidative phosphorylation, the citrate cycle, carbon metabolism, fatty acid elongation and degradation, pyruvate metabolism, biosynthesis of unsaturated fatty acids, glycolysis, and gluconeogenesis (Figure 4C). These results suggested that increased activation of ER stress and autophagy, and decreased mitochondrial function may play a critical role in cardiac dysfunction of SOCS3cko mice.

We next focused how SOCS3 deletion stimulated activation of ER stress and autophagy in cardiomyocytes in response to TAC stress. We detected several markers of ER stress and mitochondrial autophagy (also known as mitophagy) in hearts of SOCS3f/f and SOCS3cko mice after TAC. Immunoblotting analysis showed that protein levels of GRP78 (also known as BiP and HSPA5), phosphorylated dsRNA-activated protein kinase-like ER kinase (p-PERK, Thr-982), p-elF2α, cleaved ATF6 (p50 fragment), phosphorylated inositol-requiring kinase 1 (p-IRE1, Ser724), CHOP, Parkin, Nix, ATG6 (Beclin 1), and ATG5 proteins were significantly increased in SOCS3cko mice compared with SOCS3f/f animals after TAC (Figure 4D), indicating that SOCS3 is involved in controlling GRP78-mediated ER stress-mitophagy pathway in the heart after TAC.

To further confirm the effect of SOCS3 deletion on mitophagy *in vitro*, NRCMs were transfected with small interfering RNA (siRNA) against SOCS3 (siRNA-SOCS3) or scrambled control (siRNA-control), and then stained with Mtphagy Dye and Lyso Dye after 24 h of PE or vehicle treatment. Consistent with immunoblotting results, PE stimulation significantly increased induction of mitophagy (red) and mitochondrial autophagosomes (green) in siRNA-control-treated cells, but was further enhanced by siRNA-SOCS3 (Supplementary Figure 3A). Moreover, PE-induced increase of mitochondrial superoxide [as indicated by MitoSOX (red) and MitoTracker Green (green)] and reduction of mitochondrial membrane potential (ΔΨm; stained by MitoProbe JC-1) in cardiomyocytes transfected with siRNA-control was accelerated by transfection of siRNA-SOCS3 (Supplementary Figures 3B,C). Collectively, these results suggested that SOCS3 ablation in cardiomyocytes resulted in marked activation of ER stress and mitophagy leading to mitochondrial dysfunction, which may contribute to progression of cardiomyocyte apoptosis, hypertrophy, and HF.

### SOCS3 Regulated GRP78 Ubiquitination and Degradation by the Proteasome

**Figure 5:**
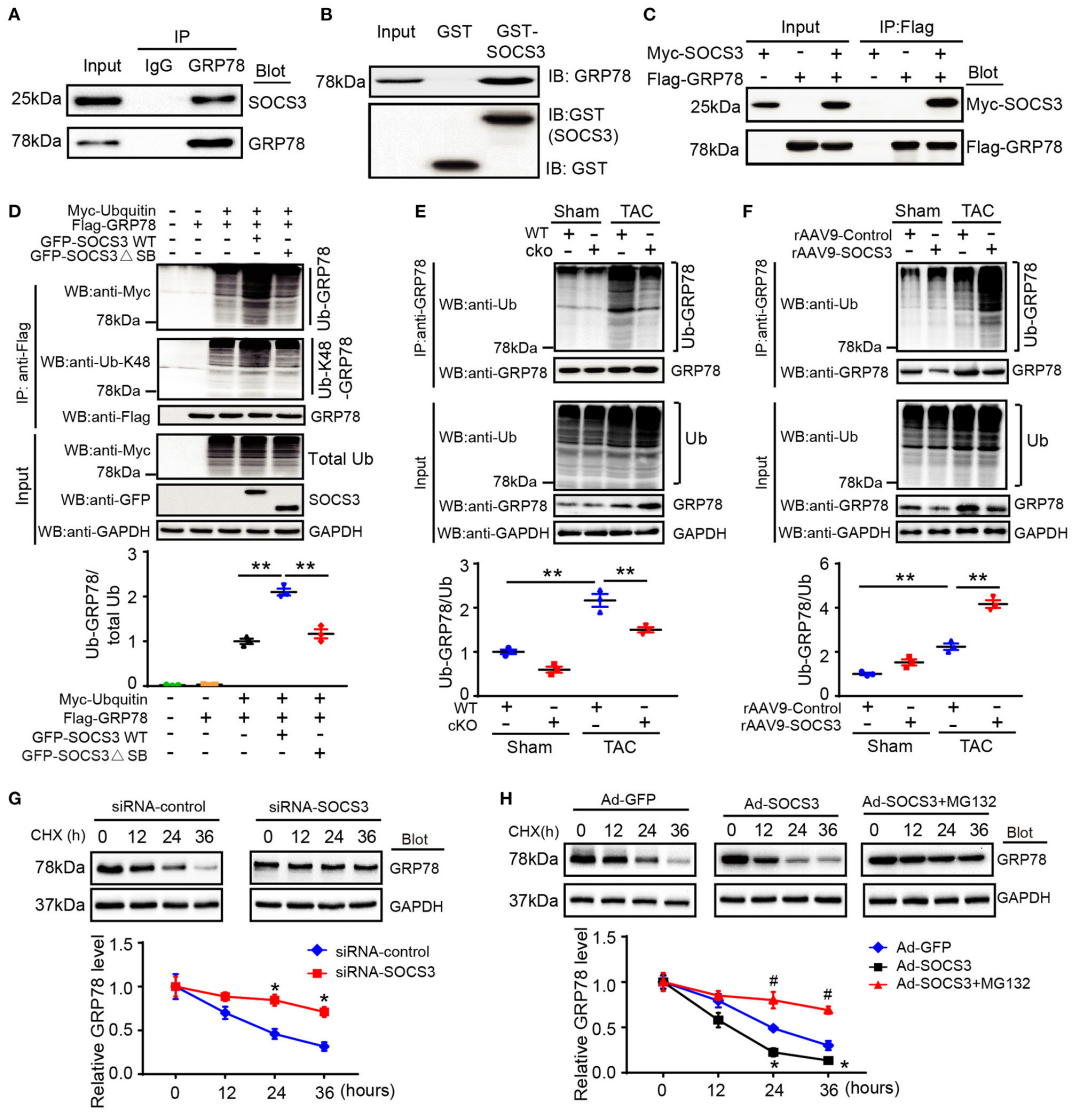
SOCS3 associated with GRP78 to promote its ubiquitination and degradation. **(A)** Endogenous SOCS3 and GRP78 protein interactions in primary cardiomyocyte lysates were evaluated by immunoprecipitation with IgG control or anti-GRP78 antibody, and then analyzed by immunoblotting (IB) with antibodies against SOCS3 and GRP78. **(B)** Protein interactions of SOCS3 with GRP78 in GST pull-down assay *in vitro*. The ability of GRP78 (top) expressed in HEK293 cells to be retained by GST or a GST-SOCS3 fusion protein was analyzed by IB after binding reactions. **(C)** HEK293 cells were transfected with the indicated plasmids. Equal amounts of protein lysates were immunoprecipitated with anti-Flag antibody and analyzed by IB with an anti-Flag (GRP78) or anti-Myc (SOCS3) antibody. **(D)** HEK293 cells were transfected with plasmids encoding Myc-tagged ubiquitin (Ub), Flag-tagged GRP78, GFP-tagged SOCS3 (WT) or its mutant (ΔSB, inactive form of SOCS3) with different combination. Equal amounts of protein lysates were immunoprecipitated with an anti-Flag antibody and analyzed by IB with anti-Myc (Ub), anti-Ub-K48 (Lys48-linked polyubiquitin) or anti-Flag (GRP78) antibodies (top). Input showed the expression of corresponding proteins in whole cell lysates (middle). Quantification of ubiquitinated GRP78 (bottom, *n* = 3). **(E)** Lysates were extracted from heart tissues of WT (SOCS3f/f) or cko (SOCS3cko) mice after Sham or TAC, and then immunoprecipitated with an anti-GRP78 antibody. IB analysis of GRP78 ubiquitination with anti-Ub or GRP78 antibody (top). Input showed IB analysis of each protein with its corresponding antibody (middle). Quantification of relative ubiquitinated GRP78 level (bottom, *n* = 3). **(F)** Lysates were isolated from heart tissues of rAAV9-Control or rAAV9-SOCS3-injected mice after Sham or TAC, and then immunoprecipitated with an anti-GRP78 antibody. IB analysis of GRP78 ubiquitination with anti-Ub or GRP78 antibody (top). Input showed IB analysis of each protein with its corresponding antibody (middle). Quantification of the relative ubiquitinated GRP78 level (bottom, *n* = 3). **(G,H)** NRCMs were infected with siRNA-control, siRNA-SOCS3, Ad-GFP, or Ad-SOCS3, and then treated with cycloheximide (CHX; 10 μM) for the indicated durations. Representative IB analysis of GRP78 and SOCS3 protein levels for each group (top), and quantification of GRP78 protein level (bottom, *n* = 3). Data are presented as mean ± SEM, and *n* represents the number of animals per group. *P < 0.05, **P < 0.01, ^#^*P* < 0.05 compared with Ad-SOCS3 + MG132.

GRP78 protein is a marker of ER stress and major ER chaperone that controls the activation of transmembrane ER stress sensors. Prompted by our results showing that GRP78 protein (Figure 4D), but not mRNA (Supplementary Figure 2E), was markedly upregulated in SOCS3cko mice compared with SOCS3f/f controls after TAC, we first examined whether SOCS3 associated with GRP78 protein in NRCMs. Co-immunoprecipitation (Co-IP) assays showed that endogenous SOCS3 protein was precipitated by an anti-GRP78 antibody, but not by a non-specific IgG control (Figure 5A). The interaction between SOCS3 and GRP78 was confirmed by an *in vitro* glutathione-S-transferase (GST) pull-down assay (Figure 5B). Furthermore, an immunoprecipitation (IP) assay was performed in human embryonic kidney (HEK) 293T cells transfected with Myc-SOCS3 and Flag-GRP78. We detected Myc-SOCS3 in the Flag-GRP78 immune complex, whereas no Myc-SOCS3 was found in controls (Figure 5C), indicating that SOCS3 interacted directly with GRP78.

To examine whether SOCS3 regulates GRP78 ubiquitination as an E3 ligase, we con-transfected HEK293T cells with plasmids encoding Myc-ubiquitin, Flag-GRP78, and either GFP-tagged wild-type (WT) SOCS3 or a catalytically inactive mutant (ΔSB: deletion of SB domain). SOCS3 overexpression (WT) significantly enhanced GRP78 ubiquitination, especially Lys48-linked polyubiquitination, whereas this effect was markedly abrogated in cells transfected with SOCS3 (ΔSB) plasmid (Figure 5D). Moreover, upon examining the effect of endogenous SOCS3 on GRP78 ubiquitination in mice, we found that TAC markedly increased GRP78 ubiquitination in SOCS3f/f mice compared with Sham groups, but this effect was remarkably attenuated in SOCS3cko hearts (Figure 5E). Conversely, the TAC-induced response was further enhanced in rAAV9-SOCS3-injected mice compared with rAAV9-ZsGreen control after TAC operation (Figure 5F).

Next, we examined the involvement of the proteasome in SOCS3-mediated degradation of GRP78, a pulse-chase assay was performed in NRCMs using cycloheximide (CHX, a eukaryote protein synthesis inhibitor) with or without MG-132 (a proteasome inhibitor). We discovered that knockdown of SOCS3 by siRNA markedly prolonged the half-life of GRP78 protein compared with the siRNA-control (Figure 5G). Conversely, SOCS3 overexpression with Ad-SOCS3 yielded GRP78 protein with a short half-life compared with Ad-GFP control, but this reduction was completely reversed by MG-132 treatment (Figure 5H), suggesting that SOCS3 promotes GRP78 degradation via the proteasome.

### GRP78 Knockdown Abrogated Pressure Overload-Induced Cardiac Hypertrophy in SOCS3cko Mice

**Figure 6:**
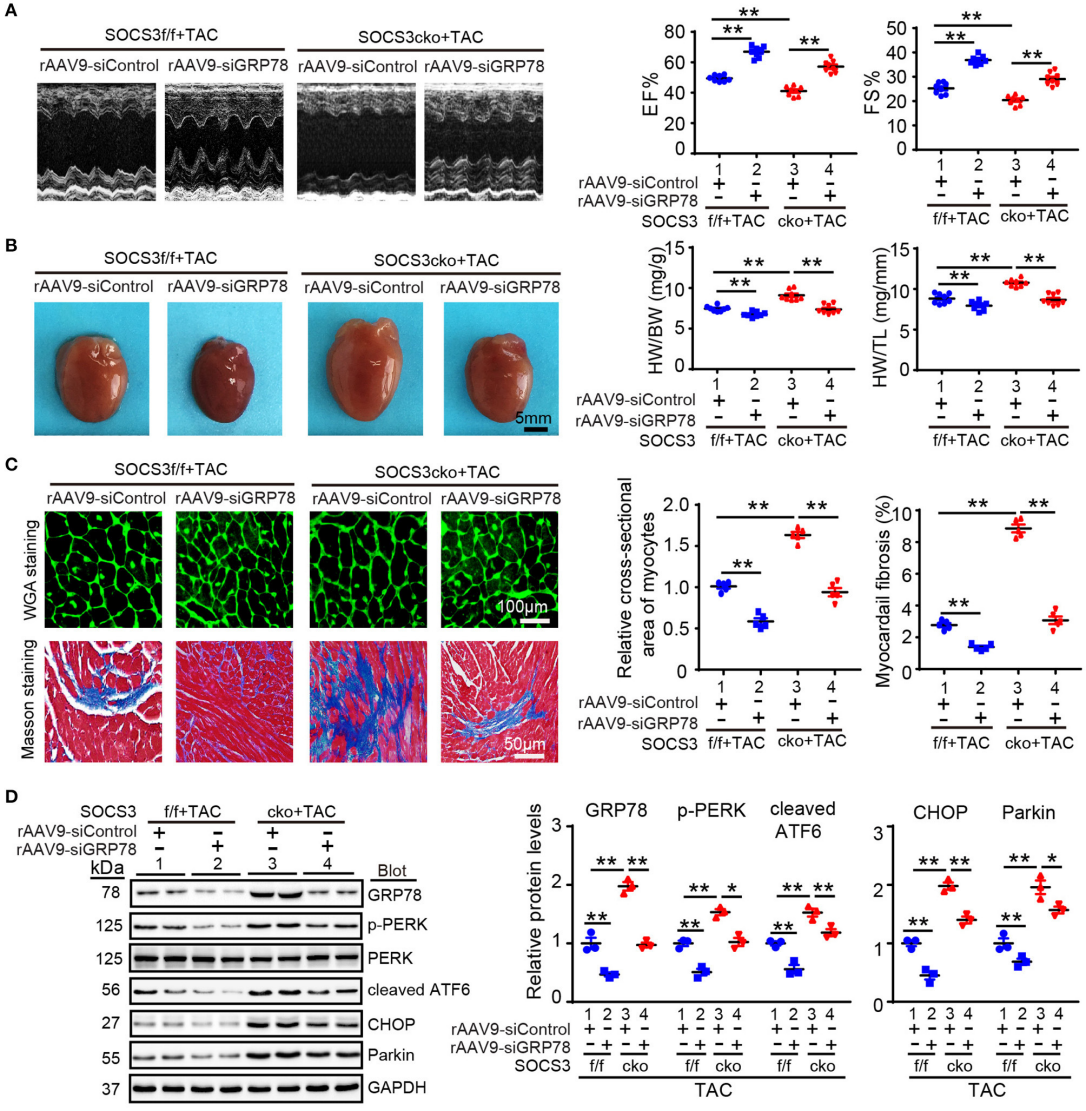
Knockdown of GRP78 by AAV9-siRNAs abolished TAC-induced cardiac hypertrophy and dysfunction in SOCS3cko mice. SOCS3f/f and SOCS3cko mice were injected with rAAV9-siControl or rAAV9-SOCS3 for 3 weeks, and then subjected to TAC surgery for an additional 4 weeks. **(A)** M-mode echocardiography of the left ventricle (left). Assessment of EF% and FS% (right, *n* = 8). **(B)** Images of hearts for size measurement (left). Quantification of HW/BW and HW/TL ratios (right, *n* = 8). Scale bar = 5mm. **(C)** FITC-labeled WGA staining of cardiac myocytes and Masson's trichrome staining of myocardial fibrosis (left). Scale bar = 100 μm. Quantification of the relative cross-sectional area of myocytes (200 cells counted per heart) and fibrotic area (right, *n* = 5). **(D)** Immunoblotting analysis of GRP78, p-PERK, PERK, cleaved ATF6, CHOP, and Parkin protein levels in heart tissues, and quantification (*n* = 3). GAPDH was used as an internal control. Data are presented as mean ± SEM, and *n* represents number of animals per group. *P < 0.05, **P < 0.01.

To assess whether GRP78 mediates cardiac hypertrophy in SOCS3cko mice after TAC, we injected SOCS3f/f and SOCS3cko mice with rAAV9-siRNA to knock down endogenous GRP78 expression. After 3 weeks of injection, all mice were then subjected to TAC for 4 additional weeks. Injection of AAV9-siGRP78 significantly downregulated GRP78 protein levels in the heart by about 45–50% compared with rAAV9-siControl (Figure 6D). Moreover, consistent with results described above (Figure 3), after 4 weeks of TAC, SOCS3cko mice showed marked cardiac dysfunction (reduced EF% and FS%), hypertrophy (increased heart size, ratios of HW/BW and HW/TL and cross-sectional areas of myocytes), and interstitial fibrosis, as well as upregulation of ANF, BNP, collagen I, and collagen III mRNA expression compared with SOCS3f/f mice after injection of rAAV9-siControl (Figures 6A–C; Supplementary Figures 4A,B, lane 3 vs. 1). Conversely, these deleterious effects were markedly reversed in SOCS3cko mice injected with rAAV9-siGRP78 (Figures 6A–C; Supplementary Figures 4A,B, lane 4 vs. 3). Similarly, rAAV9-siGRP78 injection of SOCS3f/f mice also improved TAC-induced cardiac dysfunction and hypertrophic responses compared with rAAV9-siControls (Figures 6A–C; Supplementary Figures 4A,B, lane 2 vs. 1). Correspondingly, GRP78 knockdown in SOCS3cko or SOCS3f/f mice reduced protein levels of p-PERK, cleaved ATF6, CHOP, and Parkin compared with rAAV9-siControl mice after TAC (Figure 6D). Together, these *in vivo* findings suggested that SOCS3 ablation aggravated cardiac hypertrophy and dysfunction by enhancing GRP78 and its downstream effectors.

### Inhibition of ER Stress With 4-PBA Blunted Cardiac Hypertrophy in SOCS3cko Mice Induced by Pressure Overload

To further verify the involvement of GRP78-mediated activation of ER stress in TAC-induced cardiac hypertrophy in SOCS3cko mice, SOCS3f/f and SOCS3cko mice were administered 4-PBA, a reported inhibitor of ER stress that can attenuate pressure overload-induced hypertrophy (Luo et al., 2015). As expected, 4-PBA treatment reduced GRP78 expression in both SOCS3f/f and SOCS3cko mice after TAC (Supplementary Figures 5E,F). Consistent with observations from siRNA-GRP78 knockout experiments (Figure 6), TAC-induced increases in cardiac dysfunction (reduced EF% and FS%), hypertrophy (increased heart size, ratios of HW/BW and HW/TL and cross-sectional areas of myocytes), and interstitial fibrosis were enhanced in SOCS3cko mice compared with SOCS3f/f mice (Supplementary Figures 5A–D, lane 2 vs. 1), but were significantly reduced in SOCS3cko mice treated with 4-PBA compared with vehicle (Supplementary Figures 5A–D, lane 4 vs. 2). Furthermore, 4-PBA administration in SOCS3f/f mice also showed marked cardioprotection compared with vehicle control after TAC (Supplementary Figures 5A–D, lane 3 vs. 1). The preventive effect of 4-PBA treatment on activation of ER stress and autophagy markers (p-PERK, cleaved ATF6, CHOP, and Parkin) was further confirmed in SOCS3cko and SOCS3f/f mice (Supplementary Figures 5E,F). Thus, these *in vivo* observations confirmed that the prohypertrophic effect of SOCS3 ablation resulted from activation of ER stress.

## Discussion

**Figure 7:**
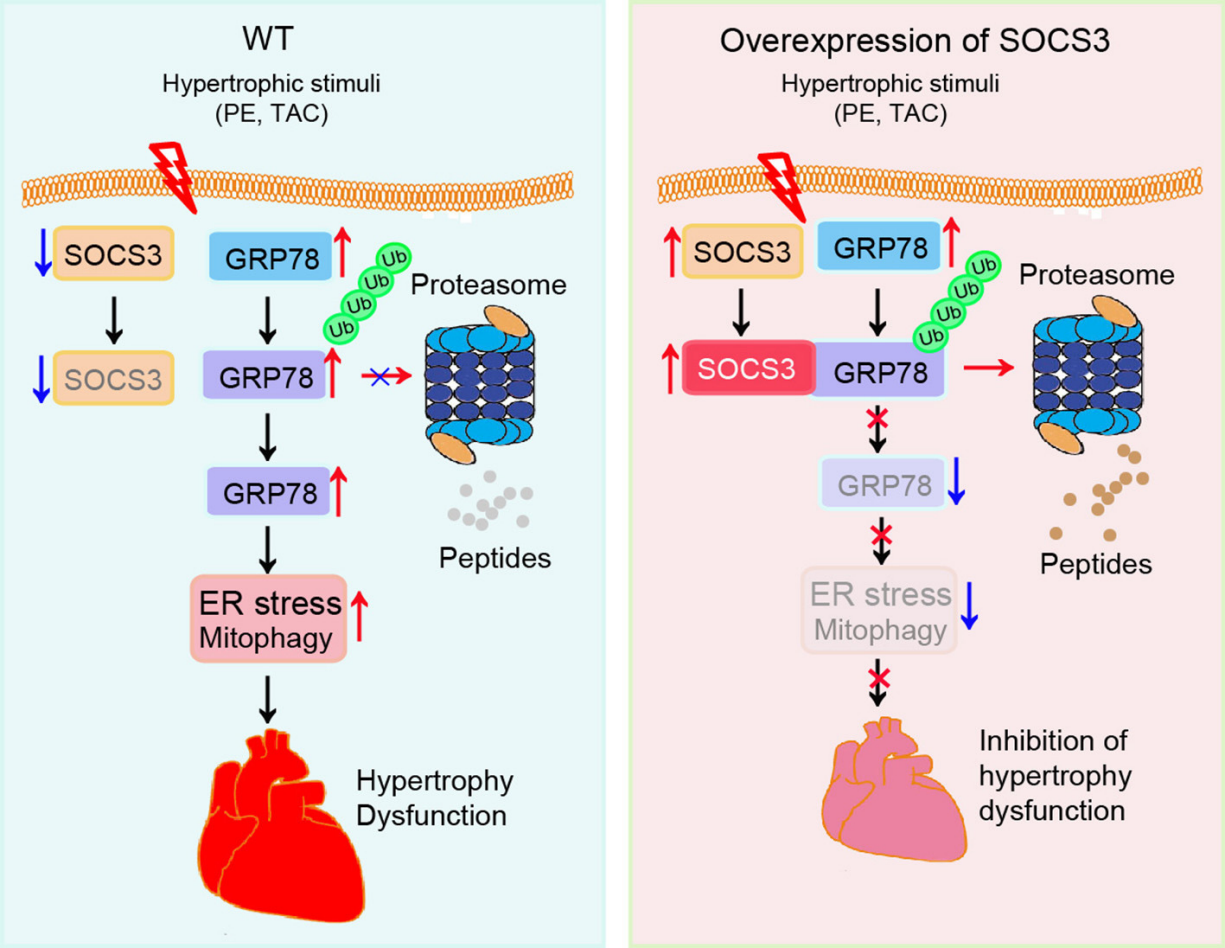
A working model of SOCS3-mediated cardioprotection in pressure overloaded mice. Pressure overload induces reduction of SOCS3 in cardiomyocytes, which in turn reduces GRP78 degradation by the proteasome and causes activation of ER stress and mitophagy, thereby leading to cardiac hypertrophy and dysfunction. Conversely, these effects are attenuated by overexpression of SOCS3.

In this study using primary cardiomyocytes, SOCS3cko mice, and rAAV9-injected wild-type mice, we identified a novel mechanism for SOCS3 to regulate cardiac hypertrophy and dysfunction through targeting of GRP78-mediated ER stress and mitophagy in a TAC-induced model. Prolonged pressure overload significantly downregulated SOCS3 expression and reduced GRP78 ubiquitination and degradation, which resulted in activation of ER stress and mitophagy, thereby leading to cardiac hypertrophy, apoptosis and dysfunction. This effect was aggravated in SOCS3cko mice, but attenuated in SOCS3-overexpression mice. Moreover, knockdown of GRP78 or inhibition of ER-stress with 4-PBA significantly attenuated ER stress and mitophagy, and restored cardiac hypertrophy and dysfunction in SOCS3cko mice (Figure 7). Thus, our novel evidence suggests that SOCS3 may be an important therapeutic target for antihypertrophic treatments.

Cardiac hypertrophy develops as an adaptive response to injury or increased workloads to maintain cardiac function. However, sustained hypertrophic remodeling contributes to progressive cardiac dysfunction and HF, although its mechanisms remain largely unknown. The ubiquitin-proteasome system is an important cellular protein degradation mechanism. Three enzymes (E1, E2, and E3) are involved in protein ubiquitination. Among them, E1 and E2 enzymes prepare the ubiquitin chain formation that is subsequently attached to the protein substrate, which is catalyzed by the E3 enzyme. During recent years, several E3 enzymes, such as F-box protein atrogin-1, muscle ring finger-1, TRAF6, and CDC20 have been reported to play roles in the development of cardiac hypertrophy through different mechanisms (Arya et al., 2004; Li et al., 2004, 2007; Ji et al., 2016; Xie et al., 2018). As an E3 ubiquitin ligase, SOCS3 targets inflammatory cytokine receptor components for proteasomal degradation. Moreover, SOCS3 is upregulated in TAC-induced hypertrophic heart (Yasukawa et al., 2001), but is reduced in failing human heart (Podewski et al., 2003; Margulies et al., 2005; Mann et al., 2010). Consistent with these data, our results also confirmed that SOCS3 expression was upregulated in hypertrophic hearts (2 weeks of TAC), but markedly downregulated in failing hearts (4 weeks of TAC, Figures 1A,B). These results suggest that SOCS3 may contribute to the transition from the adaptive cardiac hypertrophy to heart failure following pressure overload. Indeed, SOCS3 regulates cardiac hypertrophy and dysfunction partially through gp130/JAK signaling after hypertrophic stimulation (Yasukawa et al., 2001; Yajima et al., 2011). Until now, gp130 signals and myofilament Ca2^+^ sensitivity have been considered as the main pathways for SOCS3 to regulate hypertrophic response (Yasukawa et al., 2001; Yajima et al., 2011); Moreover, the present study also confirmed that SOCS3 was a critical regulator for TAC-induced cardiomyocyte hypertrophy and dysfunction *in vivo* and *in vitro* (Figures 1–3). However, whether other mechanisms, especially ER stress-mediated autophagy, participate in the cardioprotection elicited by SOCS3 was unknown.

The ER plays a crucial role in the folding of secretory and membrane proteins, as well as lipid biosynthesis and calcium homeostasis. Various types of stress such as hypoxia, ischemia, and oxidative stress, can impair ER function, leading to accumulation of misfolded and unfolded proteins – a process known as ER stress (Gotoh et al., 2011). ER stress can trigger the unfolded protein response (UPR) to induce autophagy through a number of signaling pathways (Hoyer-Hansen and Jaattela, 2007; B'Chir et al., 2013; Rashid et al., 2015). Moreover, Prolonged or severe ER stress leads to cell apoptosis, cardiac hypertrophy, and dysfunction (Yamaguchi et al., 2003; Fu et al., 2010; Minamino and Kitakaze, 2010; Yao et al., 2017). For example, tunicamycin-induced ER stress results in cardiac dysfunction, oxidative stress, apoptosis through excessive autophagy, which can be attenuated by protein tyrosine phosphatase 1B (PTP1B) ablation (Wang S. et al., 2017). Further, mitochondrial aldehyde dehydrogenase (ALDH2) protects against LPS-induced cardiac contractile dysfunction via inhibition of ER stress and autophagy via CAMKKβ/AMPK/mTOR signaling pathways (Pang et al., 2019). It is reported that autophagy induced by ER stress mainly includes the ER stress-mediated autophagy and ER-phagy. The former is characterized by the generation of autophagosomes that include protein aggregates and damaged organelles. While the ER-phagy selectively degradates ER fragments. Both of them not only have differences, but also have close connections (Rashid et al., 2015; Grumati et al., 2018). Mitophagy is an autophagic pathway that exclusively removes damaged mitochondria. Several effectors, such as Parkin and Nix, have been implicated in mitophagy activation (Yussman et al., 2002; Diwan et al., 2008; Narendra et al., 2012; Han et al., 2017). Importantly, the UPR-autophagy pathway plays crucial roles in the development of cardiovascular diseases, such as HF, hypertrophy, and ischemic heart diseases (Yussman et al., 2002; Diwan et al., 2008; Minamino et al., 2010; Narendra et al., 2012; Han et al., 2017; Zhang et al., 2018). These findings prompted us to further investigate whether ER stress-mediated autophagy or ER-phagy is involved in the cardioprotective mechanism of SOCS3 against pressure overload. Here, quantitative proteomic analysis and KEGG signaling pathway enrichment revealed that SOCS3 ablation predominantly stimulated activation of ER stress, autophagy and reduced mitochondrial function but not ER-phagy in the heart (Figures 4B,C). These effects were further confirmed by upregulation of p-PERK, p-elF2α, cleaved ATF6, p-IRE1, CHOP, Nix, Parkin, ATG6, and ATG5 proteins in SOCS3cko mice compared with SOCS3f/f animals detected by immunoblotting analysis (Figure 4D), as well as the increased mitophagy, mitochondrial superoxide production and reduced mitochondrial membrane potential in siRNA-SOCS3-transfected cardiomyocytes examined by immunostaining (Supplementary Figures 3A–C). Overall, this study provides novel evidence that SOCS3 ablation accelerates cardiac hypertrophy and dysfunction after pressure overload, possibly through activation of ER stress and mitophagy pathways. However, whether ER-phagy is involved in the development of pathological hypertrophy remains to be explored in the future.

There are at least three main ER stress sensors (PERK/eIF2α, ATF6/CHOP, and IRE1) on the ER membrane, which activate respective transcriptional cascades (ATF4, cleaved ATF6, and sXBP1, respectively) with a concomitant effect on protein translation and cell survival or death programs (Minamino et al., 2010; Gotoh et al., 2011; Senft and Ronai, 2015). GRP78 is a major ER chaperone that acts as the master regulator of the UPR through binding with inactive forms of ER-stress sensors. Increased GRP78 expression also serves as an indicator of ER stress (Gotoh et al., 2011; Wang S. et al., 2018). GRP78 is found not only in the ER lumen, but also in the cytosol, nucleus, mitochondria, and plasma membrane. Notably, intracellular GRP78 is involved in regulating ER stress-induced UPR signaling and apoptosis (Ni et al., 2011). Selective inhibition of intracellular GRP78 in the lung endothelium attenuated lipopolysaccharide-induced lung inflammatory responses (Leonard et al., 2019). Moreover, GRP78 can increase p53 nuclear localization, which in turn induces autophagy (Wang Y. et al., 2017). Interestingly, GRP78 was first reported to be increased in failing human hearts in 2004 (Okada et al., 2004). Subsequent studies confirmed upregulation of GRP78 protein levels in hearts from isoproterenol- or TAC-treated mice, as well as human dilated cardiomyopathy patients (Fu et al., 2010; Yao et al., 2017). A recent study demonstrates that ischemia/reperfusion (I/R) induces GRP78 expression in cardiomyocytes, which stimulates Akt signaling and inhibits oxidative stress leading to protection from I/R injury (Bi et al., 2018). However, the role of GRP78 in TAC-induced cardiac hypertrophy remains unknown. Here, we found that knockdown of GRP78 by siRNA significantly attenuated TAC-induced cardiac hypertrophy and dysfunction compared with siRNA control (Figure 6), indicating that GRP78 promotes cardiac hypertrophic remodeling and HF.

It has been reported that post-translational modifications such as oxidation, acetylation and ubiquitination regulate GRP78 stability and activation in different cell types (Chang et al., 2016; Kim et al., 2018; Ning et al., 2019). The E3 ubiquitin ligase GP78 (also known as AMFR and RNF45) can promote GRP78 ubiquitylation for proteasomal degradation (Chang et al., 2016). Conversely, OTUD3 interacts with and deubiquitylates GRP78, leading to its stability in cancer cells (Du et al., 2019). However, significant changes in GP78 and OTUD3 mRNA were not observed in Ang II-infused mouse hearts by microarray, suggesting that they were not involved in regulation of GRP78 in cardiomyocytes. Here, we confirmed that TAC-induced upregulation of GRP78 protein was enhanced in SOCS3cko mice (Figure 4D). Moreover, SOCS3 was found to directly associate with GRP78 (Figures 5A–C). SOCS3 overexpression increased GRP78 ubiquitination in cells and mice (Figures 5D,F), In contrast, SOCS3 inactivation (ΔSB) or knockout reduced GRP78 ubiquitination (Figures 5D,E). Further, SOCS3 knockdown enhanced GRP78 protein levels (Figure 5G). Conversely, SOCS3 overexpression reduced GRP78 protein levels, but this decrease was completely reversed by the proteasome inhibitor MG-132 (Figure 5H), indicating that SOCS3 directly interacts with and promotes GRP78 ubiquitination and degradation by the proteasome. Importantly, GRP78 knockdown or inhibition of ER stress with 4-PBA not only effectively improved TAC-induced cardiac contractile dysfunction, hypertrophy, and fibrosis, but also suppressed ER stress and mitophagy in SOCS3f/f and SOCS3cko hearts (Figures 6, 7), suggesting that GRP78-mediated ER stress plays an important role in the development of cardiac hypertrophy-related dysfunction. Overall, these results indicate that SOCS3 modulates cardiac hypertrophy and function, likely by targeting GRP78-mediated ER stress and mitophagy.

In conclusion, we discovered a novel mechanism for SOCS3 in cardioprotection, as it attenuated pressure overload-induced hypertrophic remodeling. SOCS3 most likely targets GRP78 ubiquitination for proteasomal degradation, which blocks activation of ER stress and mitophagy pathways to inhibit hypertrophic remodeling and dysfunction. Our data highlight that SOCS3 is a potential therapeutic target for treatment of hypertrophic diseases. Further studies are needed to identify activators for SOCS3, and to determine whether activation or upregulation of SOCS3 may be a therapeutic strategy for hypertrophic diseases in humans.

## Data Availability Statement

The datasets presented in this study can be found in online repositories. The names of the repository/repositories and accession number(s) can be found in the article/Supplementary Material.

## Ethics Statement

The animal study was reviewed and approved by Committee on the Ethics of Animal Experiments of Dalian Medical University.

## Author Contributions

BZ and H-HL conceived the project. SL, W-CS, Y-LZ, Q-YL, J-WL, G-RS, and X-LM performed *in vivo* and *in vitro* experiments, and analyzed the results. SL and H-HL performed and analyzed biochemical and biophysical experiments. BZ and H-HL wrote the manuscript with input from all authors. All authors contributed to the article and approved the submitted version.

## Conflict of Interest

The authors declare that the research was conducted in the absence of any commercial or financial relationships that could be construed as a potential conflict of interest.
