# Supplementary material for: SOCS3 Negatively Regulates Cardiac Hypertrophy via Targeting GRP78-Mediated ER Stress During Pressure Overload
Source: Front Cell Dev Biol. 2021 Jan 26;9:629932. doi: 10.3389/fcell.2021.629932 (PMC7874011; doi:10.3389/fcell.2021.629932)
Supplement: Supplementary file 1 [file Data_Sheet_1.docx]

**SOCS3 Negatively Regulates Cardiac Hypertrophy via Targeting GRP78-mediated ER Stress During Pressure Overload**

**Supplementary materials and methods**

**Antibodies and reagents**

The following antibodies and reagents were purchased from the indicated commercial suppliers: SOCS3 (ab16030), GRP78 (ab21685), PERK (ab79483), phospho-IRE1 (Ser724, ab48187), IRE1 (ab37073), ATF6 (ab203119), Parkin (clone PRK8, ab77924), Nix (ab8399), and GAPDH (ab8245) from Abcam (Cambridge, UK); gp130 (3732S), phospho-JAK2 (Tyr1008; clone D4A8, 8082), JAK2 (clone D2E12, 3230), phospho-STAT3 (Tyr705) (clone 3E2, 9131), STAT3 (clone 124H6, 9139), phospho-PERK (Thr980; clone 16F8, 3179), CHOP (clone L63F7, 2895), ATG6 (clone D40C5, 3495), ATG5 (clone D5G3, 9980) and K48-linkage Specific Polyubiquitin (clone D9D5, 8081) from Cell Signaling Technology (Danvers, MA); Flag (20543-1-AP), Myc (60003-2-Ig), and GST (66001-2-Ig) from Proteintech (Rosemont, IL); and ubiquitin (MAB1510) from Chemicon (Burlington, MA). Phenylephrine (PE, P1250000), cycloheximide, MG-132, and 4-PBA (P21005) were from Sigma-Aldrich (St. Louis, MO). Protein A/G Magnetic Beads for IP (B23202) were from a Pierce™ GST Protein Interaction Pull-Down Kit (21516) from Thermo Fisher Scientific (Waltham, MA). Lipofectamine® 2000 Reagent (11668-019) was from Invitrogen (Carlsbad, CA).

**Echocardiographic assessment**

Four weeks after TAC or Sham operation with administration of 4-PBA or vehicle, mice were weighed and lightly anesthetized with 1.5% isoflurane. Cardiac function and structure were then examined with echocardiography using a 30-MHz probe (Vevo 770 System; VisualSonics, Toronto, Ontario, Canada). Left ventricular (LV) anterior and posterior wall thickness (LVAW and LVPW, respectively), interventricular septum (IVS), LV end-diastolic and systolic chamber dimensions (LVESP and LVEDP, respectively), and LV end diameter (LVED) were obtained from original M-mode tracings of more than three separate cardiac cycles. LV ejection fraction (EF%) and LV fractional shortening (FS%) were calculated as previously described (Li et al., 2007; Yan et al., 2019).

**Neonatal rat cardiomyocyte isolation and culture**

Primary neonatal rat cardiomyocytes (NRCMs) were purified from Sprague-Dawley rats (aged 1–3 days) using an enzyme solution (0.04% trypsin and 0.07% type II collagenase), as previously described (Wang et al., 2018; Xie et al., 2018). Cells were cultured in Dulbecco’s Modified Eagle’s Medium with F-12 Supplement (DMEM/F12) supplemented with 10% fetal bovine serum for 16–20 hours, which was replaced with serum-free DMEM/F12 for 24 hours before experiments.

**Transfection of siRNAs**

NRCMs were transfected with siRNAs against SOCS3 (siRNA-SOCS3) or scramble sequences (siRNA-control) at 10 nM using the ON-TARGETplus Rat SOCS3 (89829) siRNA-SMART pool system (Dharmacon, Lafayette, CO) according to the manufacturer’s protocol. A 4-in-1 mix of rat SOCS3 siRNAs was used: siRNA-1 (UUAAAGUGGAGCAUCAUAC), siRNA-2 (GAAUAGAUGUAGUAAGCUC), siRNA-3 (UGACCGUUGACAGUCUUCC), and siRNA-4 (AUGCGUAGGUUCUUGGUCC), as well as control scrambled siRNA (ON-TARGETplus Non-targeting Pool Dharmacon; D-001810-10-05).

**Adenovirus and cell infection**

Adenoviruses expressing rat SOCS3 (Ad-SOCS3) or green fluorescent protein (GFP) alone (Ad-GFP) were generated using the pAdEasy system according to the manufacturer’s protocol (Hanbio Biotechnology, Shanghai, China), and as previously described (Xie et al., 2019). Sequences of SOCS3 primers were forward 5ʹ-GCTTTTCGCTGCAGAGTGAC-3ʹ and reverse 5ʹ-GGTTCCGTCGGTGGTAAAGA-3ʹ. NRCMs were infected with Ad-GFP or Ad-SOCS3 (MOI = 50) and then stimulated with PE (10 µM, Sigma-Aldrich) for 72 hours.

**Preparation and delivery of recombinant adeno-associated virus in vivo**

The mouse SOCS3 complementary DNA was constructed into the adeno-associated virus (AAV) vector (pAAV-IRES-ZsGreen). The constructed AAV-SOCS3 or control vector were transfected into 293 AAV cells (Biowit Technologies, Shenzen, China) with packaging vectors (pHelper and pAAV-RC) to generate recombinant AAV serotype 9 viruses rAAV9-SOCS3 and rAAV9-Control. For *in vivo* delivery, 100 μL of rAVV9-SOCS3 or rAAV9-Control virus particles (1 × 10^12^ viral genomes/mL) were administered to WT mice by tail vein injection. A rAAV9 construct expressing a siRNA targeting GRP78 (rAAV9-siGRP78) and corresponding control (rAAV9-siControl) were generated by Hanbio Biotechnology (Shanghai, China), as previously described (Xie et al., 2019). The siRNA target sequence for GRP78 was GGAAUGACCCUUCGGUGCA (Tamayo et al., 2011). Mice were injected with 100 μL of rAAV9-siGRP78 or rAAV9-siControl (1 × 10^11^ viral genomes/mL) by tail vein injection. Transduction efficiency of *in vivo* rAAV9 gene transfer was examined by fluorescence microscopy and immunoblotting analysis. Three weeks later, mice were subjected to TAC for 4 additional weeks.

**Immunostaining**

Immunostaining for cardiomyocytes was performed. Briefly, NRCMs were fixed in 4% paraformaldehyde for 15 min at room temperature, and then blocked in phosphate-buffered saline containing 1% bovine serum albumin and 0.5% Triton-X 100 for 30 min. Cells were subjected to immunocytochemistry using an anti-α-actinin antibody (1:100) and secondary anti-mouse antibody conjugated to TRITC or FITC (Invitrogen), as previously described (Xie et al., 2019). Cells were counterstained with DAPI to visualize their nuclei. Images of 150–200 visible cells were collected and their surface area measured using ImageJ software (NIH, Bethesda, MD).

**Mitochondrial autophagosome detection**

Mitochondrial autophagosomes (an indicator of mitophagy) were detected with a Mitophagy Detection Kit (MD01) according to the manufacturer’s instructions (Dojindo, Kumamoto, Japan). Mtphagy Dye was immobilized on intact mitochondria with a chemical bond and exhibited weak fluorescence. When mitophagy was induced, damaged mitochondria fused to lysosomes and the Mtphagy Dye emitted high levels of fluorescence. Briefly, NRCMs cultured in 24-well plates were transfected with siRNA-control or siRNA-SOCS3 for 24 hours, and then treated with PE (100 μM) or vehicle for an additional 24 hours. Cells were incubated with 100 nmol/L Mtphagy Dye working solution at 37°C for 30 min, followed by incubation with 1 µmol/L Lyso Dye working solution at 37°C for 30 min to observe co-localization of Mtphagy Dye and lysosomes. Mitophagy and fusion of mitochondria with lysosomes were observed with a fluorescence microscope (IX73; Olympus, Tokyo, Japan).

**Measurement of mitochondrial reactive oxygen species (ROS) formation**

Mitochondrial ROS levels in NRCMs were measured using MitoSOX Red superoxide indicator (M36008, Molecular Probes, Carlsbad, CA) according to the manufacturer’s instructions.

**Assessment of mitochondrial membrane potential (Δψm)**

Mitochondrial membrane potential (Δψm) of NRCMs was assessed by 5, 5’, 6, 6’-tetrachloro-1, 1′, 3, 3′-tetraethylbenzimidazole-carbocyanide iodine (JC-1; Beyotime, Shanghai, China) staining according to the manufacturer’s instructions.

**Quantitative real-time PCR analysis**

Total RNA was isolated from cultured NRCMs or fresh heart tissues using Trizol reagent (Invitrogen) according to the manufacturer’s instructions. First-strand cDNA was generated from total RNA (1–2 μg) with a reverse transcriptase enzyme mix on an S1000 PCR thermocycler (Bio-Rad, Hercules, CA). mRNA expression levels of SOCS family members (SOCS1, SOCS2, SOCS3, and CIS), atrial natriuretic factor (ANF), brain natriuretic peptide (BNP), β-myosin heavy chain (β-MHC), collagen I, and collagen III were normalized to expression of glyceraldehyde- 3-phosphate dehydrogenase (GAPDH) by the 2^-ΔΔCt^ method (Xie et al., 2018; Xie et al., 2019).

**Immunoblotting analysis**

Total proteins were purified from cultured cells or snap-frozen heart tissue using radioimmunoprecipitation assay (RIPA) buffer (PMSF : RIPA = 1:100; Solarbio Science Technology Co, Beijing, China) and quantified with a BCA Protein Assay Kit according to the manufacturer’s instructions. Cell extracts (40–50 μg) were subjected to sodium dodecyl sulfate-polyacrylamide gel electrophoresis (SDS-PAGE), transferred to polyvinylidenedifluoride membranes (Bio-Rad), and then probed with the indicated primary antibodies at 4°C overnight. The following day, membranes were incubated with horseradish peroxidase-conjugated secondary antibodies (1:3000) for 1 hour at room temperature, as previously described (Xie et al., 2018; Xie et al., 2019). All blots were developed using a chemiluminescent system (Gel-Pro 4.5 Analyzer; Media Cybernetics, Rockville, MD). Signal intensities were quantified with ImageJ software and normalized to GAPDH (an internal control).

**Plasmids and transient transfection**

Flag epitope-tagged GRP78 was obtained from Sino Biological Inc (Beijing, China). Myc-tagged wild-type SOCS3 and its inactive form (△SB, deletion of SB domain) were kind gifts from Dr. Wenchang Sun. Transient transfection of plasmids was carried out in HEK293T cells using Lipofectamine 2000 Reagent.

**Immunoprecipitation assay**

Immunoprecipitation (IP) was performed with Protein A/G Magnetic Beads for IP according to the manufacturer’s protocols as previously described (Xie et al., 2018; Xie et al., 2019). Briefly, cultured cells or fresh heart tissues were lysed with buffer containing 50 mM Tris, 150 mM NaCl, 0.1%–0.5% NP40 (pH 7.5), and protease inhibitor cocktail (Thermo Fisher Scientific) on ice for 20 min. For precipitation, 2 μg of primary antibody (Ab) and 20 μL of A/G magnetic beads (Amersham Biosciences, Little Chalfont, UK) were added to protein samples at 4°C for 2 hours. Samples were washed three times with buffer containing 50 mM Tris-HCl (pH 7.5), 500 mM Sodium chloride, 0.1% NP-40, and 0.05% sodium deoxycholate. Bead complexes were eluted with 20–40 μL of 2× SDS-PAGE loading buffer. Samples were boiled for 5 minutes and then subjected to immunoblotting using specific primary and secondary antibodies to recognize targeted proteins. Precipitated proteins were subjected to immunoblotting with specific primary and secondary antibodies. Blot intensities were quantified as described above.

**GST pull-down assay**

The cDNA encoding rat SOCS3 was cloned into the pGEX-4T1 vector. GST-SOCS3 fusion proteins were expressed in *Escherichia coli* BL21 cells. The empty vector (pGEX-4T1) expressing GST alone was used as a negative control. GST pull-down assay was performed using a Pierce GST Protein Interaction Pull-Down Kit. The purified GST-SOCS3 fusion protein was immobilized on a Pierce-Spin Column according to the manufacturer’s instructions. GRP78 was then blotted as described above.

**In vivo ubiquitylation assay**

An *in vivo* ubiquitination assay was performed according to a previously described protocol (Li et al., 2007). Protein lysates from HEK293T cells or fresh heart tissues were precipitated and detected by immunoblotting analysis using appropriate antibodies (including Flag, Myc, GFP, ubiquitin, SOCS3, and GRP78).

**Pulse-chase analysis**

A pulse-chase assay was performed to measure the half-life of expressed GRP78 proteins, as previously described (Xie et al., 2018; Xie et al., 2019). Briefly, NRCMs were infected with an adenovirus expressing GFP alone, GFP-SOCS3, siRNA-control, or siRNA-SOCS3 for 24 hours. Protein lysates were prepared at indicated time points after the addition of cycloheximide (CHX, 20 μM) or CHX plus MG-132 (10 μM). Levels of endogenous GRP78 protein were detected by immunoblotting analysis with an anti-GRP78 antibody.

**Supplementary Figures**

**
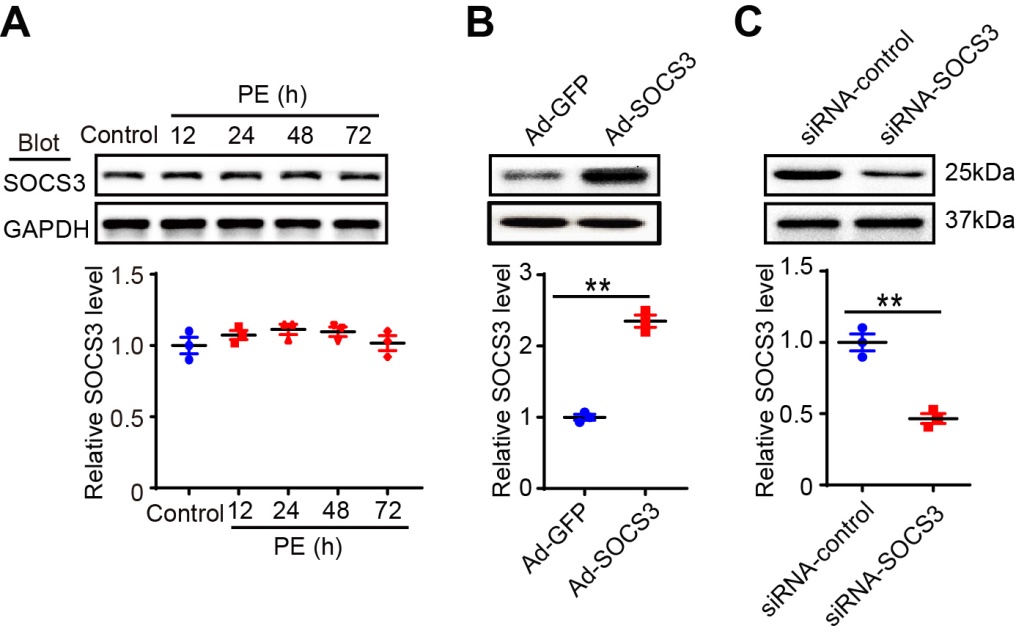
**

**Supplementary Figure S1** Analysis of the SOCS3 protein levels in neonatal rat fibroblasts and cardiomyocytes. **(A)** Immunoblotting analysis of SOCS3 protein levels in neonatal rat fibroblasts treated with PE (100 μmol/L) for different time points (upper), and quantification of the relative SOCS3 protein level (lower, *n* = 3). **(B)** Immunoblotting analysis of SOCS3 protein expression in neonatal rat cardiomyocytes (NRCMs) infected with adenovirus vector expressing GFP (Ad-GFP) or SOCS3 (Ad-SOCS3) for 24 hours (upper). Quantification of the relative SOCS3 protein level (lower, *n* = 3). **(C)** Immunoblotting analysis of SOCS3 protein expression in NRCMs infected with siRNA-control or siRNA-SOCS3 for 24 hours (upper). Quantification of the relative SOCS3 protein level (lower, *n* = 3). Data are presented as mean ± SEM, and *n* represents number of samples per group. ***P* < 0.01.


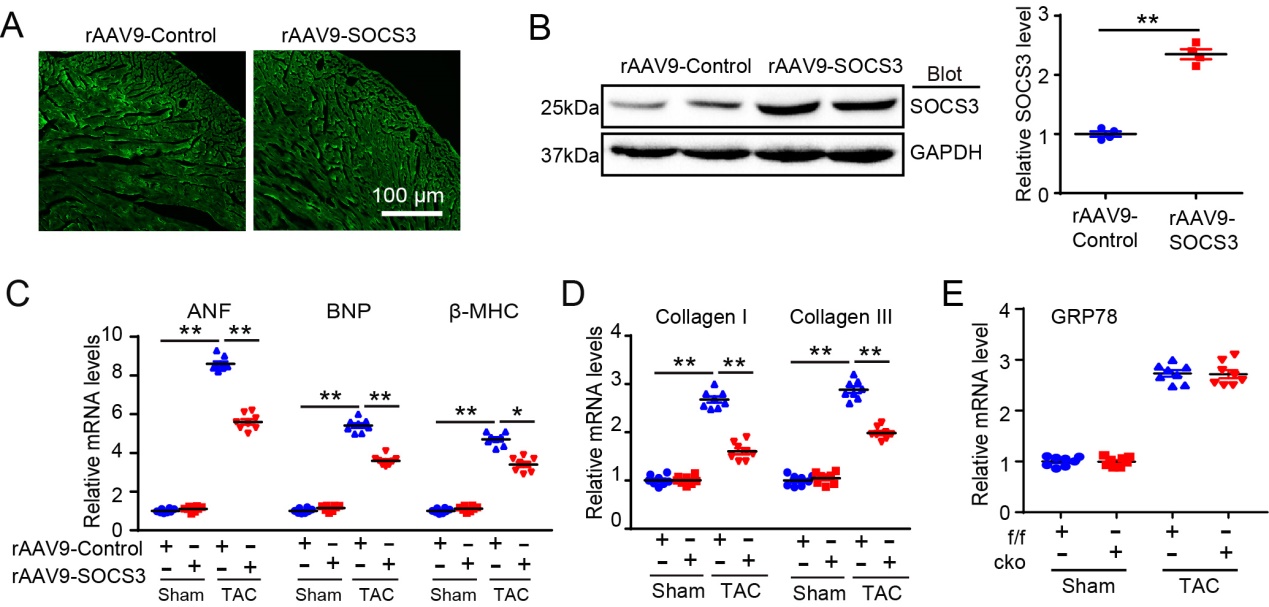


**Supplementary Figure S2** Efficiency of rAAV9 gene injection in mice. **(A)** Representative immunofluoresence examination of ZsGreen protein in mouse heart infected with rAAV9- ZsGreen Control or rAAV9-SOCS3 for 3 weeks. Scale bar 100 μm. **(B)** Immunoblotting analysis of SOCS3 protein level in the hearts (left). GAPDH as an internal control. Quantification of the relative SOCS3 protein level (right, *n* = 4). **(C,D)** qPCR analysis of hypertrophic markers (ANF, BNP and β-MHC) and fibrotic markers (Collagen I and III) mRNA expression in the heart tissues of rAAV9-ZsGreen Control- or rAAV9-SOCS3-injected mice after 4 weeks of Sham or TAC (*n* = 8). **(E)** WT (SOCS3f/f) and cardiomyocyte-specific SOCS3 knockout mice (SOCS3cko) were subjected to Sham or TAC operation for 4 weeks. qPCR analysis of GRP78 mRNA level in the hearts (n = 4). Data are presented as mean ± SEM, and *n* represents number of samples per group. **P* < 0.05, ***P* < 0.01.


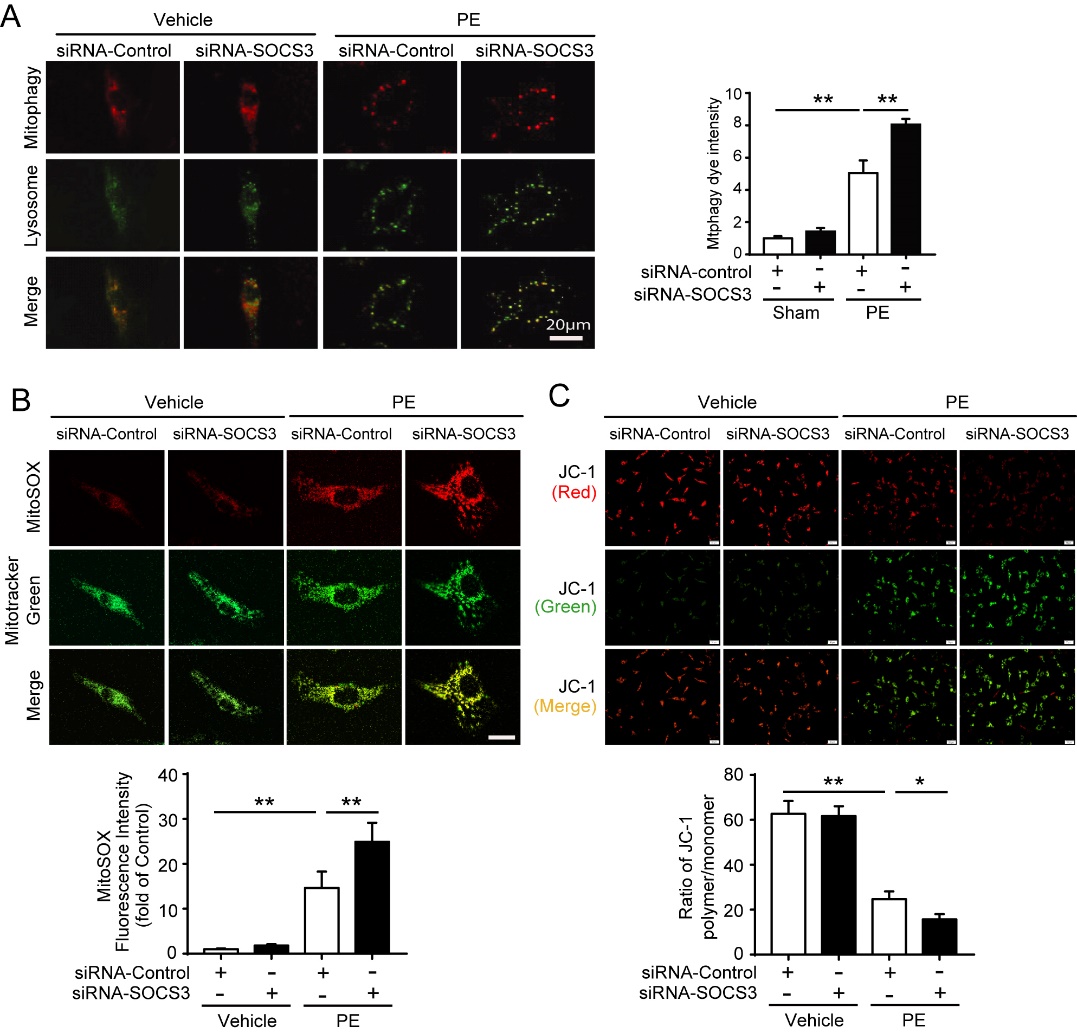


**Supplementary Figure S3** Analysis of mitophagy, superoxide level and mitochondrial membrane potential in primary cardiomyocytes after PE treatment. (**A**) Neonatal rat cardiomyocytes (NRCMs) were infected with siRNA-Control or siRNA-SOCS3 for 24 hours and then treated with PE (100 μmol/L) for 72 hours. Images of double immunostaining (red indicates mitophagy, green indicates lysosomes) of NRCMs. Quantification of Mtphagy Dye intensity (right). Scale bar = 20 μm. (**B**) The level of mitochondrial superoxide in NRCMs was detected with MitoSOX (red) and MitoTracker Green (green) (upper). Quantification of the relative MitoSOX fluorescence intensity (lower, 150 cells counted per experiment, *n* = 3). (**C**) NRCMs were infected and treated as in **A**. The mitochondrial membrane potential (△Ψm) in NRCMs was stained with MitoProbe JC-1 (upper). Quantification of the relative polymer (red) to monomer (green) (lower, 150 cells counted per experiment, *n* = 3). Data are presented as mean ± SEM. **P* < 0.05, ***P* < 0.01.


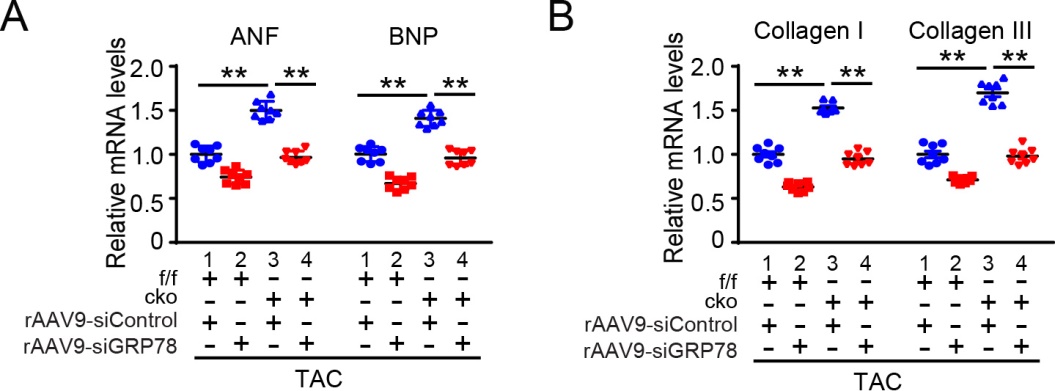


**Supplementary Figure S4** Knockdown of GRP78 by rAAV9-siRNAs blunted TAC-induced upregulation of cardiac hypertrophic and fibrotic markers in SOCS3cko mice. SOCS3f/f and SOCS3cko mice were injected with rAAV9-siControl or rAAV9-SOCS3 for 3 weeks, and then subjected to TAC surgery for an additional 4 weeks. **(A)** qPCR analysis of ANF and BNP mRNA levels in the hearts (*n* = 8). **(B)** qPCR analysis of Collagen I and Collagen III mRNA levels in the hearts (*n* = 8). Data are presented as mean ± SEM, and *n* represents the number of animals per group. ***P* < 0.01.


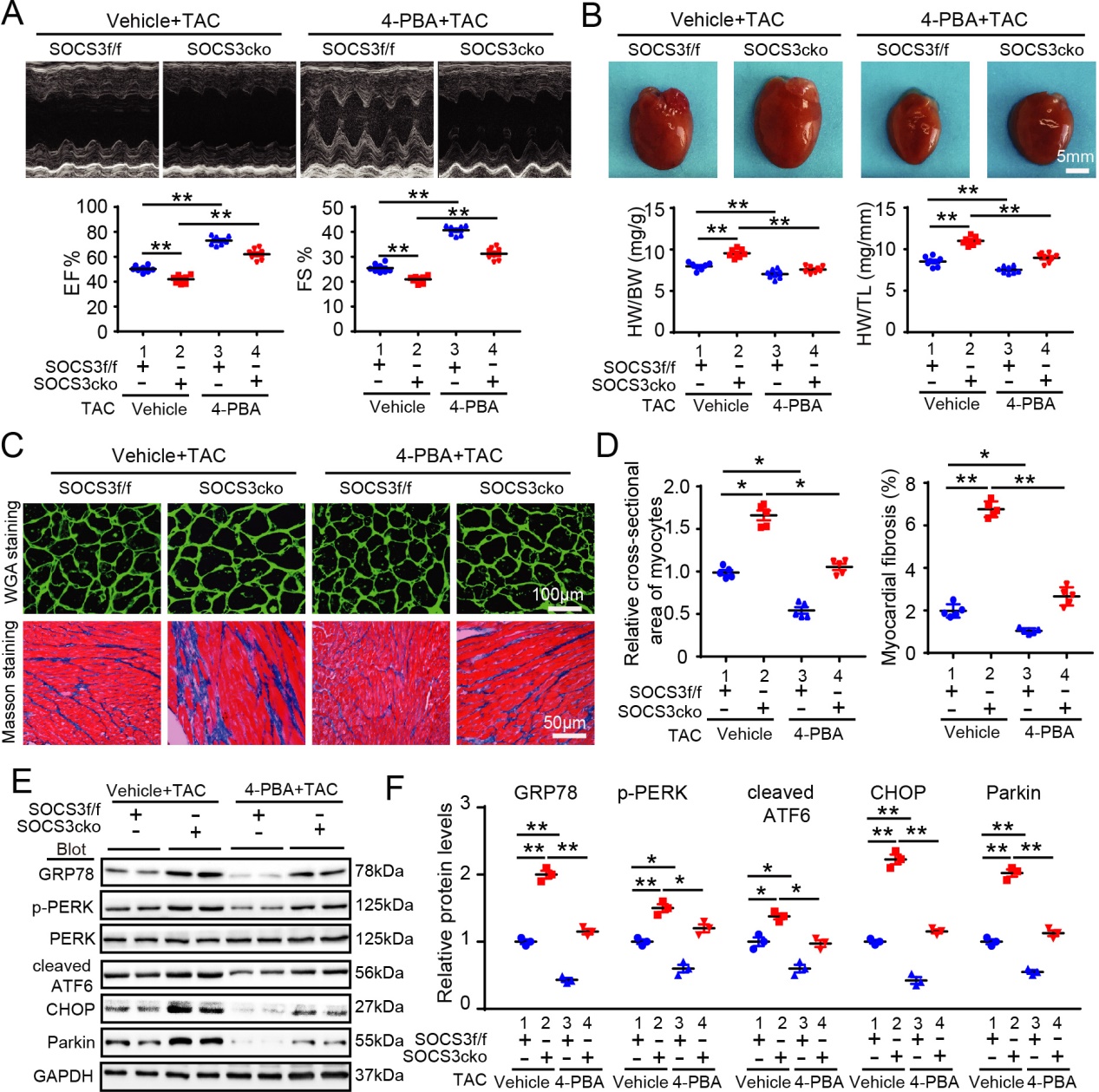


**Supplementary Figure S5** Inhibition of the ER stress by 4-PBA abrogated TAC-induced cardiac hypertrophy and dysfunction in SOCS3cko mice. **(A)** SOCS3f/f and SOCS3cko mice were treated TAC surgery with vehicle or 4-PBA (20 mg/kg/day) for 4 weeks. M-mode echocardiography of the left ventricle (left). Assessment of EF% and FS% (right, *n* = 8). **(B)** Representative images of hearts for size measurement (left). Analysis of HW/BW and HW/TL ratios (right, *n* = 8). Scale bar = 5 mm. **(C)** FITC-labeled WGA staining of cardiac myocytes and Masson’s trichrome staining of myocardial fibrosis (left). Scale bar = 100 μm. **(D)** Quantification of the relative cross-sectional area of myocytes (200 cells counted per heart) and fibrotic area (right, *n* = 5). **(E,F)** Immunoblotting analysis of GRP78, p-PERK, PERK, cleaved ATF6, CHOP, and Parkin protein levels in heart tissues, and quantification of the relative protein levels (*n* = 3). GAPDH was used as an internal control. Data are presented as mean ± SEM, and n represents number of animals per group. **P* < 0.05, ***P* < 0.01.

**References**

Li, H.H., Willis, M.S., Lockyer, P., Miller, N., McDonough, H., Glass, D.J., et al. (2007). Atrogin-1 inhibits Akt-dependent cardiac hypertrophy in mice via ubiquitin-dependent coactivation of Forkhead proteins. *J. Clin. Invest.* 117(11)**,** 3211-3223. doi: 10.1172/JCI31757.

Tamayo, A.G., Slater, L., Taylor-Parker, J., Bharti, A., Harrison, R., Hung, D.T., et al. (2011). GRP78(BiP) facilitates the cytosolic delivery of anthrax lethal factor (LF) in vivo and functions as an unfoldase in vitro. *Mol. Microbiol.* 81(5)**,** 1390-1401. doi: 10.1111/j.1365-2958.2011.07770.x.

Wang, L., Zhang, Y.L., Lin, Q.Y., Liu, Y., Guan, X.M., Ma, X.L., et al. (2018). CXCL1-CXCR2 axis mediates angiotensin II-induced cardiac hypertrophy and remodelling through regulation of monocyte infiltration. *Eur. Heart J.* 39(20)**,** 1818-1831. doi: 10.1093/eurheartj/ehy085.

Xie, X., Bi, H.L., Lai, S., Zhang, Y.L., Li, N., Cao, H.J., et al. (2019). The immunoproteasome catalytic beta5i subunit regulates cardiac hypertrophy by targeting the autophagy protein ATG5 for degradation. *Sci. Adv.* 5(5)**,** eaau0495. doi: 10.1126/sciadv.aau0495.

Xie, Y.P., Lai, S., Lin, Q.Y., Xie, X., Liao, J.W., Wang, H.X., et al. (2018). CDC20 regulates cardiac hypertrophy via targeting LC3-dependent autophagy. *Theranostics* 8(21)**,** 5995-6007. doi: 10.7150/thno.27706.

Yan, X., Zhang, Y.L., Zhang, L., Zou, L.X., Chen, C., Liu, Y., et al. (2019). Gallic Acid Suppresses Cardiac Hypertrophic Remodeling and Heart Failure. *Mol. Nutr. Food Res.* 63(5)**,** e1800807. doi: 10.1002/mnfr.201800807.
